# Systematic review of thyroid function in *NKX2-1*-related disorders: Treatment and follow-up

**DOI:** 10.1371/journal.pone.0309064

**Published:** 2024-10-28

**Authors:** Beatriz Carmona-Hidalgo, Estefanía Herrera-Ramos, Rocío Rodríguez-López, Laia Nou-Fontanet, José C. Moreno, Juan Antonio Blasco-Amaro, Juliane Léger, Juan Darío Ortigoza-Escobar, NKX2-1-Related Disorders Guideline Working Group

**Affiliations:** 1 Health Technology Assessment Area-AETSA, Andalusian Public Foundation for Progress and Health (“Fundación Progreso y Salud”–“FPS”), Seville, Spain; 2 Evaluation Unit (SESCS), Canary Islands Health Service (SCS), Santa Cruz de Tenerife, Spain; 3 Canary Islands Health Research Institute Foundation (FIISC), Las Palmas de Gran Canaria, Spain; 4 Network for Research on Chronicity, Primary Care, and Health Promotion (RICAPPS), Madrid, Spain; 5 Department of Child Neurology, Movement Disorders Unit, Institut de Recerca Sant Joan de Déu, Barcelona, Spain; 6 Thyroid Molecular Laboratory, Institute for Medical and Molecular Genetics (INGEMM), Research Institute of Paz University Hospital (IdiPAZ), Madrid, Spain; 7 U-753 The Rare Diseases Networking Biomedical Research Centre (CIBERER), Instituto de Salud Carlos III, Madrid, Spain; 8 European Reference Network on Rare Endocrine Conditions (Endo-ERN), Amsterdam, The Netherlands; 9 Endocrinology-Diabetology Department, Assistance Publique-Hôpitaux de Paris, Robert Debre´ University Hospital, Reference Center for Growth and Development Endocrine Diseases, Paris, France; 10 Université Paris Cité, NeuroDiderot, Institut National de la Santé et de la Recherche Médicale (INSERM 1141), Paris, France; 11 U-703 Centre for Biomedical Research on Rare Diseases (CIBER-ER), Instituto de Salud Carlos III, Barcelona, Spain; 12 European Reference Network for Rare Neurological Diseases (ERN-RND), Tübingen, Germany; PGIMER: Post Graduate Institute of Medical Education and Research, INDIA

## Abstract

**Background:**

*NKX2-1*, a crucial transcription factor in thyroid, lung, and brain development, is associated with rare disorders featuring thyroid dysfunction, neurological abnormalities, and respiratory symptoms. The primary challenge in managing *NKX2-1*-related disorders (*NKX2-1-*RD) is early diagnosis of the genetic defect and treating specific endocrine disorders. Levothyroxine (LT4) serves as the standard hypothyroidism treatment, with required dosages influenced by the severity of the individual’s disorder, which varies widely among affected individuals.

**Objectives:**

This systematic review aims to assess the effectiveness of LT4 treatment in *NKX2-1*-RD and explore optimal dosing strategies. The primary focus is on the challenges associated with the prompt diagnosis of genetic defects, rather than the established treatment protocols for individual endocrine failures.

**Methods:**

Adhering to PRISMA guidelines, the review includes 42 studies involving 110 genetically confirmed *NKX2-*1-RD patients with hypothyroidism. The study investigates congenital hypothyroidism as the most prevalent endocrine alteration, along with gestational and overt hypothyroidism. The administration of LT4 treatment, dosages, and patient responses are analyzed.

**Results:**

Among the findings, congenital hypothyroidism emerges as the predominant endocrine alteration in 41% of patients. Notably, LT4 treatment is administered in only 10% of cases, with a mean dose of 52 μg/day. The variability in initiation and dosage is likely influenced by the age at diagnosis. Positive responses, characterized by TSH adjustments within normal ranges, are observed in 11 monitored patients.

**Conclusions:**

Early detection of congenital hypothyroidism is emphasized for timely LT4 initiation. Challenges in standardization are highlighted due to the variability in clinical manifestations and diagnostic procedures across *NKX2-1*-RD cases. While this review provides valuable insights into thyroid and pituitary disease treatment, limited details on LT4 treatment represent a significant study limitation. Key reporting points for future case studies are proposed to enhance the understanding and management of *NKX2-1*-RD hypothyroidism.

## Introduction

*NKX2-1*, located in chromosome 14q13.3 (OMIM*600635, previously known as *TTF-1*) is a critical transcription factor involved in the development and function of the thyroid, lung, and brain. Genetic abnormalities in *NKX2-1* have been associated with a spectrum of rare disorders characterized by a wide range of clinical manifestations, including thyroid dysfunction, neurological abnormalities, and respiratory problems. Patients with *NKX2-1*-related disorders (*NKX2-1*-RD, OMIM#610978) or Benign Hereditary Chorea (BHC) often present with complex endocrine manifestations, such as congenital overt or subclinical hypothyroidism and pituitary deficiency. These diverse presentations pose significant challenges in diagnosis, treatment, and long-term management [1–3]. Thyroid-stimulating hormone (TSH) represents the most sensitive indicator of thyroid dysfunction. Congenital hypothyroidism (CH) typically causes low thyroxine (T4) and free T4 (fT4) levels with elevated TSH levels. In compensated or subclinical hypothyroidism, serum T4 remains normal while the TSH level is elevated. Early screening is essential for identifying infants affected by these conditions. Neonatal screening programs (NBS) and early treatment initiation (prior to 2 weeks of life after birth) can prevent intellectual deficits and improve neurodevelopmental outcomes [4, 5].

*NKX2-1*-RD patients are advised to follow standardized treatment guidelines for managing endocrine manifestations, particularly congenital hypothyroidism. Levothyroxine (LT4) is considered the preferred treatment for hypothyroidism. The recommended initial dose is adjusted based on the severity of the disease, the age, and the weight of the patient. The LT4 starting dose is typically in the range of 3–15 μg/kg/day, considering the spectrum of CH in the patient. Infants with severe CH (low levels of fT4 in serum ≤5 pmol/L- in combination with elevated TSH ≥20 mU/L-) should be treated with the highest starting dose of LT4 (10–15 μg/kg/day). On the other hand, infants with mild CH (fT4 ≥10 pmol/L- and elevated TSH) should be treated with the lowest initial dose of LT4 (∼10 μg/kg/day) [6]. Regular monitoring of serum fT4 and TSH levels should be conducted at frequent intervals in children to ensure a favorable disease prognosis. An initial LT4 dose can normalize TSH in 2 weeks and serum fT4 in 3 days of therapy [6–8]. The key hurdle is ensuring early diagnosis to institute mandatory follow-up and proactively screen for additional endocrine alterations such as pituitary deficiency. Untreated or poorly managed endocrine abnormalities in *NKX2-1*-RD can lead to serious health consequences, affecting the physical and cognitive development of affected individuals, particularly premature newborns due to impaired thyroid activity [9]. Early detection, appropriate treatment, and close follow-up are crucial to optimizing patient outcomes and quality of life. In navigating the complexities of LT4 replacement therapy dosing and hormone level monitoring, it is worth acknowledging that these aspects are already well-established, with existing guidelines for reference [10]. While awareness of *NKX2-1-*RD is growing, there is still a gap in the literature concerning the optimal treatment strategies and long-term follow-up plans for patients, particularly those dealing with endocrine issues, primarily hypothyroidism. A deep review of the literature addressing these aspects will provide valuable insights to guide clinical decision-making and future research efforts.

The European Reference Network for Rare Neurological Disorders (ERN-RND) together with the European Network for Rare Endocrine Conditions (Endo-ERN) are working for the first time on the development of a Clinical Practice Guideline for patients with *NKX2-1-*RD as part of the ERN Guidelines Program. In this systematic review, we aim to analyze the existing evidence on the effectiveness of LT4 treatment in different endocrine presentations of *NKX2-1*-RD and to appraise the well-established dosing strategies for LT4 replacement therapy. Additionally, we seek to evaluate the frequency and necessity of hormone level monitoring during follow-up to guide clinicians in their decision-making process.

## Methods

This study covers the systematic review of a research question conducted following the Preferred Reporting Items for Systematic Review and Meta-Analyses (PRISMA) statement [11]. PRISMA checklist is specified in **S1 File**.

### Research question

The main PICO question (acronym for Population-Intervention-Comparator-Outcome) of the initial systematic review was: *What sort of endocrinologic follow-up is recommended to monitor the onset of endocrinologic diseases in NKX2-1-related disorders*?. It was designed to serve as a guide for the literature search. Later, it was divided into three research questions to cover all the aspects related to the detection, diagnosis, treatment, and follow-up of endocrine alterations in *NKX2-1*-RD. The questions related to initial screening and diagnosis have been addressed in a prior study. Here, we focus on the following question: *What are the best procedures for treatment and follow-up of endocrine diseases in patients with NKX2-1-related disorders*?. The protocol of the systematic review was previously registered in the PROSPERO repository with the identification CRD42022341011.

### Eligibility criteria

Specific inclusion criteria were employed to select the relevant studies. The population criteria included patients of all ages with genetic confirmation of *NKX2-1*-RD (pathogenic variants in *TTF-1/NKX2-1* or deletion in the 14q13.3 chromosome) and hypothyroidism. Non-human studies and patients without BHC genetic confirmation were excluded. Given the rare condition, any comparator was used. The intervention of interest to treat endocrine alterations was defined as LT4 treatment. The follow-up interventions to monitor the evolution of the disease were the quantification of thyroid hormone levels in serum and hearing tests and ultrasound examinations as additional clinical evaluations. Any other type of intervention related to hypothyroidism but not to *NKX2-1*-RD was excluded. The outcomes based on treatment were the effectiveness of LT4 treatment and adverse effects. The outcomes related to follow-up were established as neurodevelopmental improvements, puberty development, bone, metabolic, and cardiovascular health, patient and professional education, quality of life, and self-management in adulthood. The study designs included were primary studies, systematic reviews, and randomized controlled trials (RCTs). Narrative reviews, conference articles, editorials, letters to the editor, and studies whose full text could not be obtained were excluded.

### Search strategy

To review the scientific evidence, a literature search was carried out in reference databases such as PubMed, Embase, the Cochrane Library, MEDLINE (Ovid), PsyncINFO, CINAHL, the TRIP medical database, and the Health Technology Assessment (HTA) database. Subject-specific databases were included to retrieve data about rare disease resources such as Orphanet, EURORDIS, NORD, RARE-Best Practices, and Gene Reviews. We searched clinical trial registries, such as CENTRAL and the International Clinical Trials Registry Platform (ICTRP). The initial search covered the period from January 2002 to May 2022, with an update in July 2023. The records that have been collected since 2002 pertain to the initial discovery of the pathogenic variants of the *NKX2-1* gene as the cause of BHC [12]. Both controlled language (descriptors) and free terminology were used to search for studies. The initial strategy was carried out in MEDLINE (Ovid) and later adapted to each database’s syntax **(S2 File)**.

### Study screening and data extraction

The references from the searches were imported into the software application Covidence (https://www.covidence.org/) for the screening process, where the duplicates were removed. Two authors independently filtered the references according to the inclusion and exclusion criteria (BCH and JDOE). The first selection was carried out by title and abstract, and the second one by full-text screening. Both reviewers reached an agreement in the case of discrepancies. The disagreements were resolved by a third researcher (JL). The data were extracted by two independent authors (BCH and JDOE) and recorded in Excel spreadsheets. Specific details of the studies (author, year of publication, study type, and location) and specific clinical data of each patient (age, sex, genetic tests, thyroid, neurological, and respiratory affections, age at onset of hypothyroidism, thyroid gland alteration, treatment regimen, effectiveness of treatment, adverse events, and follow-up) were extracted. Missing data points were labeled with ’NA’ (Not Available) to indicate their absence in the dataset. These data points were not included in the final analysis. For analyses requiring complete datasets, cases with missing data were excluded listwise (complete-case analysis) to avoid introducing bias due to imputation. Sensitivity analyses were conducted to assess the impact of excluding these cases on the study’s overall findings.

### Quality assessment

The quality of the studies was assessed by two authors (BCH and JDOE) in an independent way. Disagreements between individual judgments were resolved by discussion to reach a consensus. The quality assessment was carried out using specific tools for observational studies, as the included references were classified as case reports, case series, and cohort studies. The tool developed by Murad et al. [13] was used for case reports and case series. This tool allows us to assess the methodological quality of the studies with a low body of evidence through eight items categorized into four domains (selection, ascertainment, causality, and reporting). According to the criteria of the tool, the quality of articles was rated by summing the scores of the eight binary responses (0 = high risk of bias, 1 = low risk of bias) into an aggregate score. The score ranges from 0 (very poor quality) to 8 (very high quality) [14]. The items of ascertainment, causality, and reporting were adapted to the specific clinical setting of the research question. The Newcastle-Ottawa Scale (NOS) was used to assess the cohort studies [15]. It uses three categories (selection, exposure, and comparability) with eight items to identify good-quality decisions using stars. One star can be given to each item in the selection (maximum of 4 starts) and outcome (maximum of 3 stars) categories, and a maximum of two for comparability. No studies were excluded on low-quality grounds. The Grade of Recommendation, Assessment, Development, and Evaluation System (GRADE) was used to evaluate the quality of the evidence provided by the studies. This system sets four categories (high, moderate, low, and very low), considering five factors: risk of bias, inconsistency, indirectness, imprecision, and publication bias [16].

## Results

### Study selection

**Fig 1:**
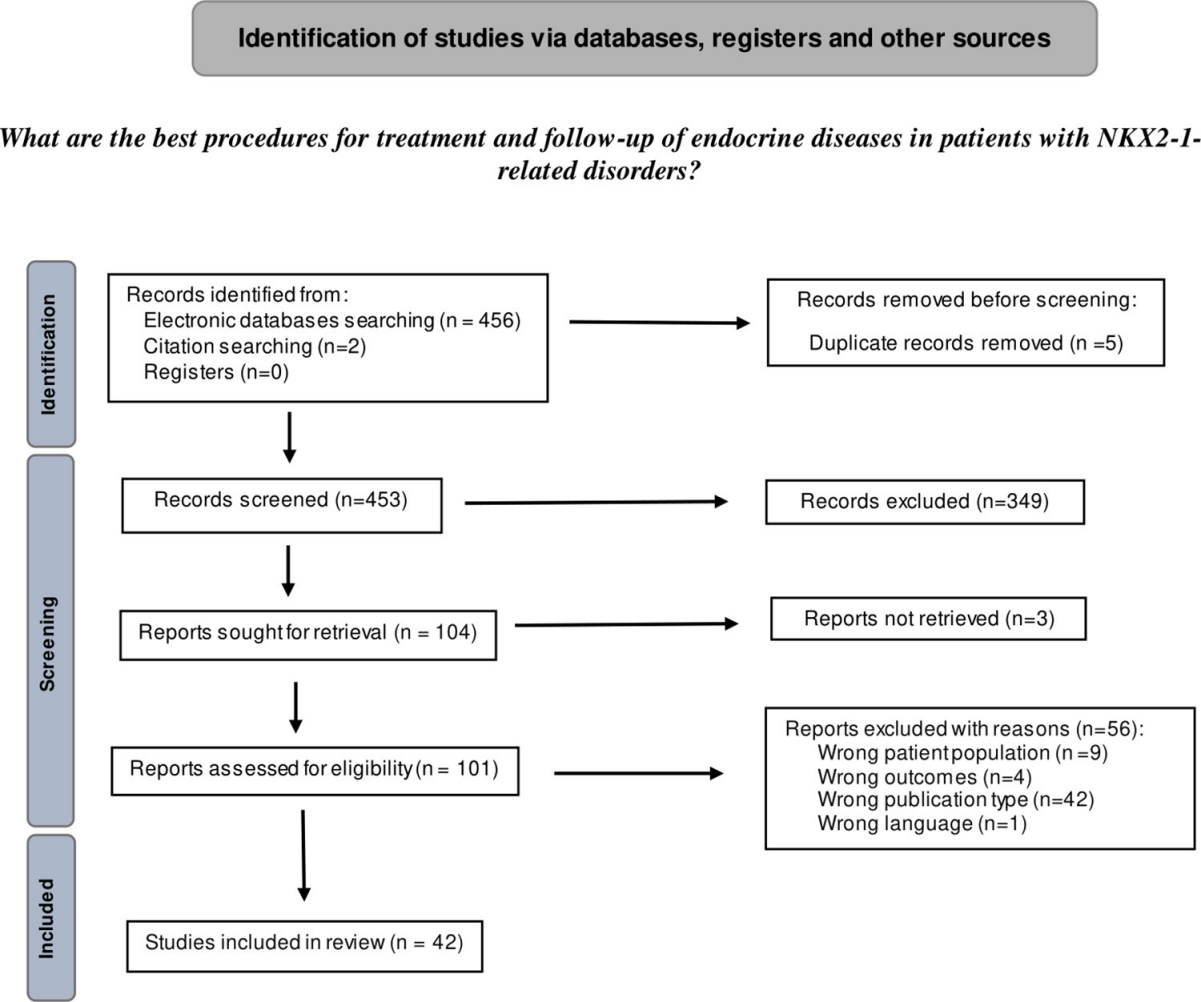
PRISMA flowchart for treatment and follow-up of endocrine diseases in patients with *NKX2-1-*RD. Illustration of the study selection process followed according to the Preferred Reporting Items for Systematic Review and Meta-Analyses (PRISMA) guidelines. The flowchart depicts the number of records identified from the initial search, duplicates removed, and the number of studies excluded at each screening stage based on title and abstract filters, as well as full-text screening.

In the initial search, 458 records were identified. After eliminating duplicates, 453 potentially relevant studies remained. Title and abstract filters were applied, resulting in 104 studies for further assessment. Based on inclusion and exclusion criteria, 42 studies were included after full-text screening. The PRISMA flowchart in **Fig 1** illustrates the screening process. The list of included and excluded articles with the reasons for exclusion, after full-text screening, is shown in **S3 File.** The excluded articles were classified according to the first criterion that did not fit the PICO format of the defined research question.

### Study characteristics

**Table 1** shows the 42 studies analyzed. Among these, 50% were case series (n = 21), 47.6% were case reports (n = 20), and only 2.4% constituted cohorts (one study falling into this category). The rarity of the clinical condition under investigation resulted in the absence of randomized control trials or systematic reviews. Geographically, 61.9% were conducted in Europe (n = 26), 19% in America (n = 8), 16.7% in Asia (n = 7), and only one in Australia.

**Table 1:** List of included references for treatment and follow-up of endocrine diseases in patients with *NKX2-1-*RD. This table provides details such as author, year, location, and study type of the final included articles.

Author, year	Location	Study type	Author, year	Location	Study type
**Asmus, 2005**	Germany	Case series	**Makretskaya, 2018**	Russia	Cohort
**Balicza, 2018**	Hungary	Case series	**Maric, 2020**	Bosnia-Herzegovina	Case report
**Barnett, 2012**	Australia	Case report	**Moya, 2018**	Spain	Case report
**Barreiro, 2011**	Spain	Case report	**Nagasaki, 2008**	Japan	Case report
**Carré, 2009**	France	Case series	**Nattes, 2017**	France	Case series
**Delestrain, 2023**	France	Case series	**Parnes, 2019**	United States	Case series
**Doyle, 2004**	United States	Case series	**Prasad, 2019**	United Kingdom	Case report
**Ferrara, 2012**	United States	Case series	**Provenzano, 2016**	Italy	Case series
**Fons, 2012**	Spain	Case Report	**Safi, 2017**	United States	Case series
**Galambos, 2010**	United States	Case report	**Salerno, 2014**	Italy	Case report
**Gonçalves, 2019**	Portugal	Case report	**Salvado, 2013**	Spain	Case series
**Gras, 2012**	France	Case series	**Salvatore, 2010**	Italy	Case series
**Gu, 2020**	China	Case report	**Santos-Silva, 2019**	Portugal	Case report
**Hayasaka, 2018**	Japan	Case series	**Shiohama, 2018**	Japan	Case report
**Kharbanda, 2017**	United Kingdom	Case report	**Tanaka, 2020**	Japan	Case report
**Kleinlein, 2011**	Germany	Case report	**Thust, 2022**	United Kingdom	Case series
**Koht, 2016**	Norway	Case series	**Trevisani, 2022**	Italy	Case report
**Krude, 2002**	Germany	Case series	**Uematsu, 2012**	Japan	Case series
**Kumar, 2014**	United States	Case series	**Veneziano, 2014**	United Kingdom	Case series
**Li, 2023**	China	Case series	**Villafuerte, 2018**	Spain	Case report
**Lynn, 2020**	United States	Case report	**Villamil-Osorio, 2021**	Colombia	Case report

### Patients demographics

The study included a total of 110 patients diagnosed with *NKX2-1*-RD and confirmed hypothyroidism. 53.6% were female, with a mean age of 15.2 years, ranging from 28 days to 64 years. The pathogenic variants and *NKX2-1* deletions were frameshift 41 (37.3%), missense 38 (34.6%), *NKX2-1* gene deletion 15 (13.6%), splicing 12 (10.9%), and nonsense variants 4 (3.6%).

In 84 patients (76.3%), sequencing, including next-generation sequencing (NGS) and whole exome sequencing (WES), was used to identify the *NKX2-1* gene variants. Other methods employed include comparative genomic hybridization (CGH) array (11 patients, 10%), fluorescence in situ hybridization (FISH) (2 patients, 1.8%), and karyotyping analysis (1 patient, 0.9%). In 12 patients (11%), the specific genetic technique used for deletion or variant identification was not reported. *De novo* mutations were identified in 31.8% of patients (n = 35).

CH, or subclinical hypothyroidism, was the most common endocrine alteration, affecting 60% of the patients (n = 66). Two pregnant women showed gestational hypothyroidism [17, 18]. 36.4% (n = 40) had overt hypothyroidism. At least one patient had euthyroid-sick syndrome [19].

Some patients showed other endocrine alterations, along with hypothyroidism. Two patients had dysfunctional pituitary glands [20, 21], with one having a mildly hypoplastic pituitary gland and a Rathke’s cyst and the other presenting with an empty sella. In another study, a cystic pituitary mass was detected along with low prolactin levels and macroadenoma [22]. A female CH patient had undetectable gonadotrophins, neonatal transient hyperinsulinism, growth hormone and cortisol deficiencies. Neonatal transient hyperinsulinism manifested on the 18th day, characterized by a biochemical finding of insulin at 10 mU/L accompanied by hypoglycemia necessitating treatment with diazoxide and chlorothiazide [23]. A second patient had a cortisol deficiency [19]. A male patient with hypothyroidism, low testosterone, and luteinizing hormone had severe hypogonadism as an adult [21]. A second male patient with subclinical hypothyroidism had growth hormone and serum insulin-*like* growth factor-I (IGF-I) deficiency [23].

*NKX2-1-*RD affects the endocrine, neurological, and respiratory systems. In this study, 60.9% (n = 67) reported having the complete triad of the disorder. 28.2% of the patients (n = 31) only had endocrine and neurological alterations, while endocrine and respiratory involvement were reduced to 8.2% (n = 9). In three patients, only endocrine affectation was reported without more details (2.7%). It should be highlighted that chorea and hypotonia were the most dominant neurological disorders diagnosed in the first years of life. Neonatal respiratory distress was the most common postnatal respiratory alteration. *NKX2-1*-RD patients often have dysmorphic features, congenital anomalies, or developmental delay. The severity of the dysfunction determines whether it is detected in the first year or in childhood. Several patients in our study had learning impairments and behavioral disorders [24, 25]. Facial dysmorphism, palatal cleft, dental agenesis, fetal finger pads, a prominent forehead, and flaring nostrils were most visible in young patients [20, 26–28]. Some brain malformations included corpus callosum agenesis and dysgenesis, mild cerebellar atrophy, glioblastoma, hippocampus dysmorphism, and microcephaly [24, 29–33]. One patient had supratentorial leukoencephalopathy [29], and another patient had multiple birth defects [34]. 6 (5.4%) study participants died from severe clinical complications [18, 19, 35–38]. A detailed summary of the baseline characteristics of the included patients is provided in **S4 File**.

### Treatment regimen for endocrine alterations in patients with *NKX2-1-*RD

In our systematic review, only 12 patients (10.9%) from 11 studies were reported to receive LT4 treatment after confirming CH [27, 28, 33, 39–42], subclinical hypothyroidism [24, 30], and overt hypothyroidism [43, 44]. Eight of these patients had altered neonatal screening, and four had overt hypothyroidism. In patients with overt hypothyroidism, the age of treatment initiation was 18 months, 21 months, 2 years, and 4 years. Screening centers had different hypothyroidism threshold values, causing uncertainty and disagreement. Many studies did not provide detailed information on neonatal screening or subsequent diagnostic tests, resulting in fluctuations in the initiation and dosage of LT4 treatment among patients and studies. Based on these data, the mean and median LT4 dose at the start of treatment were 52 and 37.5 μg/day, respectively. The mean age of treatment was 10.7 months, with a median of 35 days (range: 8 days to 4 years). Three patients received weight-based doses [30, 40, 44], with variations in the initial dose due to differences in age at onset and severity of hypothyroidism. The patients who had an altered neonatal screening started the LT4 treatment at neonatal age, in the first month of life, to normalize the hormone levels rapidly [28, 33, 39, 40]. Among the patients with a normal neonatal screening or those in whom it was not reported, they were diagnosed later, so the LT4 treatment started at older ages with the consequent physical and clinical alterations [30, 31, 41, 44]. Additional treatment regimen details can be found in **S5 File**.

### Follow-up of endocrine alterations in patients with *NKX2-1-*RD

The follow-up period after hypothyroidism confirmation was reported in 11 of the 12 patients who received LT4 treatment. They constitute only 10% of the total number of patients in this study. TSH levels were used most commonly for monitoring thyroid function alterations. They were monitored from the beginning of treatment with LT4 for regular periods of time to control thyroid hormone fluctuations. The discontinuation of LT4 treatment and dose adjustments to regulate thyroid hormones were common strategies. Due to the complexity of *NKX2-1-*RD, the frequency of the follow-up also depended on the resolution of the hypothyroidism and the severity of other clinical manifestations such as neurological and/or respiratory alterations. In the 11 patients who received LT4 treatment and had a follow-up period, they showed positive responses. The dose of LT4 was gradually increased or diminished in order to normalize thyroid function, along with regular monitoring.

As stated in the preceding paragraph, we found important differences and a lack of data in the initial dosage of treatment. However, some patients reached a similar dose during the follow-up period, although the starting dose showed differences [30, 42, 44]. In two patients, the treatment was suspended at 4 years [27] and 18 months [30] of age because of a permanent stabilization of TSH levels. However, data on treatment effectiveness were not reported in a study [33]. During the thyroid hormone monitoring, a patient showed thyroid function normalization at day 30 of life, although the LT4 dose was gradually increased during childhood to maintain thyroid levels in the normal range. However, he continued with a bone development delay detected at 2 years. The remaining follicle-stimulating hormone, luteinizing hormone, and testosterone hormone values were adequate for the developmental age [28].

Similarly, a male patient was diagnosed with subclinical hypothyroidism along with growth hormone (GH) deficiency [30]. During the follow-up, thyroid function was normalized due to LT4 treatment, but neurological alterations and growth delays persisted. LT4 and GH treatments were administered to the patient, who showed clinical improvements. A neurological treatment was not considered due to the decrease in the severity of symptoms. Notably, one patient showed psychomotor delay and hypotonia, although euthyroidism was maintained after LT4 therapy [41]. This patient experienced discontinuation of treatment for four weeks, leading to disproportionate increases in TSH levels due to resistance. A second patient suffered frequent drop attacks if LT4 treatment was suspended, although he experienced stable euthyroidism. The drop attacks resolved upon treatment restoration [44]. However, it is important to acknowledge that these episodes may potentially be associated with the onset of chorea in an infant learning to walk. While the link between the disappearance of drop attacks and LT4 treatment is viewed as highly speculative, it is worth noting that the resolution of drop attacks could also be attributed to levodopa treatment. No studies reported adverse events related to the treatment. As anticipated, LT4 treatment improved stuttering, fatigue, and weight gain [39]. To track treatment efficacy and cover all follow-up procedures in *NKX2-1*-RD patients, hearing and ultrasound tests were included in the research question. Finally, this review found no evidence for these interventions. Detailed information on patient monitoring is provided in **S5 File**.

### Complementary treatments for neurological and respiratory alterations in patients with *NKX2-1-*RD

A total of 40% of the patients received treatment for chorea and other movement disorders. Levodopa-carbidopa was the most commonly used treatment, showing effectiveness in 57% of the patients. However, side effects were reported in 24% of the patients, including dyskinesia, falls, sedation, dizziness, fatigue, drowsiness, drooling, freezing of gait, digestive intolerance, insomnia, nervousness, and asthma attacks [17, 34, 45–47]. Regarding respiratory alterations, 14% of the patients received treatment. The main treatments included surfactant for newborns with respiratory distress, antibiotics for infections, and oxygen. These treatments were effective in 12% of cases. Side effects were reported in 9% of patients and were primarily associated with prolonged steroid use, leading to conditions such as Cushing’s syndrome, adrenal insufficiency, osteoporosis, hypokalemia, and reduced bone density. Additionally, one patient presented with neutropenia as a result of hydroxychloroquine administration [18].

### Quality assessment of the included studies

In this systematic review, the quality of each study was evaluated on the basis of the scoring systems. According to the assessment tools, 73.2% (n = 30) of case series and case reports were considered to have poor methodological quality, 19.5% (n = 8) medium quality, and 7.3% (n = 3) good quality. The cohort study was assessed as of poor quality [48]. The scores for each item and the total score for the studies are listed in **S6 File**.

### Quality assessment of the evidence

Due to the limited number of evaluated patients and the variability of the data, a quantitative synthesis of the results was not feasible in this systematic review. The inadequate study design downgrades the initial quality assessment. The estimation of the risk of bias, inconsistency, indirectness, imprecision, and publication bias was made qualitatively. Firstly, the data showed high variability due to the different treatment regimens among patients in dosage, age at initiation, follow-up time, and dose adjustment. Moreover, the lack of reported data on the severity of thyroid alterations and the hormone levels at neonatal screening and/or diagnosis tests makes it difficult to establish LT4 treatment ranges in correlation with the previous patients’ parameters. Secondly, the indirectness of the study was considered low as the available evidence was directed at the initial research question. The imprecision of the results was high due to the small sample size and the lack of reporting of results of interest. The literature searches were designed to be sensitive enough to retrieve studies related to the research question. It is possible that some of them have been lost, but the results reported are similar in terms of not giving information about the values of the treatment dose, the follow-up, the age at onset, and the effectiveness of the treatment. Despite this, they show that they consistently align with the current available evidence on treatment strategies for *NKX2-1*-RD. Regarding publication bias, it was not possible to perform an objective assessment due to the lack of information from unpublished studies. Therefore, the quality of the evidence collected in this systematic review can be considered low for the previous factors analyzed.

## Discussion

Through a complete analysis of the available evidence, this study presents a comprehensive overview of the current treatment and follow-up protocols for endocrine diseases in individuals with *NKX2-1-*RD for the first time. Our analysis highlights the significance of timely and accurate neonatal screening for CH to facilitate early detection and prompt initiation of LT4 treatment. For all causes of hypothyroidism, including but not limited to *NKX2-1*-RD, the implementation of standardized guidelines for LT4 dosing, incorporating factors such as weight, is essential. This standardized approach ensures the maintenance of optimal hormone levels and contributes to improved treatment outcomes. Furthermore, long-term follow-up should be established for patients with confirmed hypothyroidism and *NKX2-1*-RD to monitor the effects of LT4 treatment on thyroid function, adjust dosages if needed, and address potential side effects to ensure overall clinical success. Crucially, infants diagnosed with even mild CH should not undergo a withdrawal period, a decision contingent upon the timely availability of a genetic diagnosis conducted early in the diagnostic process and an ongoing follow-up protocol.

We conducted a study involving a cohort of 110 patients, following an exhaustive screening process of 453 scientific articles. These articles were identified through a sensitive search strategy implemented across a wide range of databases using specific terms relevant to the field. Given the rare nature of the disease and the search period from 2002, when the responsible gene for the disorder was identified [12], the number of studies and patients included in our analysis was considered optimal. The patients in our cohort exhibited a diverse range of age, genetic variants, geographical locations, and clinical manifestations, contributing to the external validity of our findings. To ensure internal validity, we established strict inclusion criteria, only selecting patients with a confirmed genetic diagnosis of the disease and concurrent hypothyroidism. Rigorously trained and independent reviewers performed the screening, data extraction, and quality assessment processes following a standardized methodology.

CH represents the main endocrinological manifestation in patients with *NKX2-1*-RD. This condition is typically characterized by elevated levels of TSH and low T4 or fT4 concentrations, which can be detected through neonatal screening, enabling early intervention with thyroid hormone replacement therapy using LT4 [4, 49]. The manifestation of hypothyroidism may vary, with some cases being permanent while others may resolve during childhood or adulthood. *NKX2-1*-RD exhibit a diverse range of phenotypes, ranging from CH detected in infancy to cases diagnosed later in childhood or adulthood, as well as compensated hypothyroidism that does not necessitate treatment [50].

The definition of hypothyroidism and its confirmation threshold vary among different screening centers, lacking a clear consensus. Depending on the time after birth and the screening test technology, accepted TSH threshold values range from 5 to 12 mU/L in the UK and up to 10 mU/L in Spain [40]. An initial TSH level >50 μU/L is often indicative of permanent CH, while TSH levels between 20 and 49 μU/L may be false positives or indicate transient hypothyroidism [2, 51]. For subclinical hypothyroidism, TSH ranges from 6 to 20 mU/L. Notably, many studies in this systematic review did not provide sufficient information regarding neonatal screening or subsequent diagnostic tests for confirmation of hypothyroidism in *NKX2-1*-RD patients to allow the start of proper treatment. The follow-up of patients with *NKX2-1*-RD and hypothyroidism is more common during childhood and early adolescence, when symptoms of *NKX2-1*-RD become evident from birth [52].

For cases of *NKX2-1*-RD and hypothyroidism where formal recommendations cannot be made and, at present, there is no available curative treatment, guidance from current guidelines or consensus statements should be followed. As highlighted in the clinical consensus update of ENDO-RND, LT4 is strongly recommended as the preferred medication for the treatment of CH. Initiation of treatment should occur promptly, ideally within two weeks after birth or immediately after confirmatory thyroid function testing, once CH is detected through a second routine screening test in neonates. This recommendation applies as a general rule for all patients with CH, encompassing considerations beyond *NKX2-1*-RD.

We recommend the follow-up and monitoring of treatment through the evaluation of fT4 and TSH levels in accordance with age-specific reference intervals. In patients with *NKX2-1*-RD and CH, the initial clinical and biochemical follow-up assessment should be conducted within 1 to 2 weeks after the initiation of LT4 treatment. Subsequent evaluations, both clinical and biochemical, should occur every 2 weeks until serum TSH levels are fully normalized. After complete normalization of TSH, the evaluation frequency can be reduced to once every 1 to 3 months until the age of 12 months. From 12 months to 3 years of age, the evaluation frequency can be further lowered to every 2 to 6 months, and later, from 3 years until growth completion, evaluations should take place every 3 to 6 months. Patients diagnosed with *NKX2-1*-RD and CH should adhere to the aforementioned recommendations and follow current knowledge [53, 54].

While isolated hyperthyrotropinemia is sometimes considered to have minimal effects on health, recent knowledge on its long-term cardiovascular and metabolic consequences [55] leads the panel of experts to advise levothyroxine treatment for TSH elevation in the case of NKX2-1-RD, based on the genetic and therefore permanent nature of TSH elevations in these cases. Furthermore, the mechanism for hyperthyrotropinemia in *NKX2*-1 haploinsufficiency is the low expression of the TSH receptor, and neonatal hyperthyrotropinemia due to resistance to TSH (by heterozygous mutations in the TSHR gene) are usually (87%) treated in neonates [56]. For adults, we suggest individualized therapeutic approaches, even in cases with borderline (normal to slightly low) free thyroxine (FT4) levels within the reference range [57, 58].

The observation of transient hypothyroidism [40] in some patients with subsequent stabilization of TSH levels, even without treatment, raises an intriguing aspect. While there is not a clear pathophysiological basis to corroborate this finding specifically in patients with *NKX2-1*-RD hypothyroidism, it prompts a crucial consideration for the management of CH cases, particularly those associated with *NKX2-1* pathogenic variants or deletions. Re-evaluation of thyroid function is imperative for all patients with CH and an intact thyroid gland, as emphasized in the latest guidelines by Trotsenburg et al. [6]. However, the long-term evolution of thyroid function in *NKX2-1*-RD patients remains incompletely understood. Given the potential for mild hypothyroidism in these cases, routine re-evaluation should be conducted between 6 months and 3 years of age, regardless of the genetic testing results, to prevent unnecessary treatment. While genetic testing results may take time to become available in many countries, it is essential to proceed with re-evaluation within the specified timeframe to ensure timely management. Regarding the timing of genetic result availability in CH patients, it is common practice to wait for genetic confirmation before re-evaluation. However, it is important to note that definitive withdrawal of treatment is uncommon, even in cases where genetic defects are known to be present. Typically, levothyroxine treatment is maintained, even in cases of euthyroid hyperthyrotropinemia around 6 mU/L, as withdrawal may pose risks. Therefore, a cautious approach is warranted in managing *NKX2-1*-RD hypothyroidism, considering both clinical and genetic factors in decision-making.

Due to the detection of hypothyroidism, including transient cases during pregnancy, special monitoring is recommended for pregnant women with *NKX2-1-*RD. Specific recommendations for this group can be found in other guidelines [6]. Physiologically, during pregnancy, thyroid hormone experiences significant changes due to the influence of various hormones and other physiological factors. These changes include an increase in thyroid hormone requirements due to the heightened metabolic demands of both the mother and the fetus. Maternal thyroid dysfunction during pregnancy is associated with neurodevelopmental impairment in the offspring. At this juncture, it is crucial to underscore the significance of screening strategies for managing gestational hypothyroidism during pregnancy and prior to conception [59].

*NKX2-1-*RD is a syndrome that affects the functionality of the neurological and respiratory systems, along with the endocrinological one. The literature suggests a potential role for LT4 as a treatment option for drop attacks in cases of *NKX2-1*-RD with euthyroidism [44]. However, it is important to note that this observation is highly speculative, and further studies are necessary to better understand and confirm the effectiveness of LT4 in managing drop attacks in this specific context. On the other hand, proper patient and caregiver education on recognizing potential side effects of treatment and the importance of adherence to LT4 therapy can significantly enhance treatment compliance and overall health outcomes.

In our study, we found that 59% of the patients presented with combined neurological, thyroid, and respiratory conditions. Additionally, 29% exhibited endocrine and neurological alterations, while 7% showed endocrine and respiratory dysfunction. These findings align with previous literature reports, which state that approximately 50% to 57% of patients with *NKX2-1* gene abnormalities may display the complete triad of the syndrome, and both hypothyroidism and central nervous system abnormalities are observed in 30% of patients [52, 60].

The main limitation of this study pertains to the absence of crucial information on the treatment and follow-up of patients, resulting in challenges in comprehensively interpreting the data and arriving at conclusive findings. Surprisingly, only a small proportion of patients with congenital hypothyroidism (CH) in reviewed papers had detailed information regarding levothyroxine (LT4) treatment, while data regarding the type of treatment received by the remaining patients were often lacking. We hypothesize that this observation may be attributed to the underlying assumption made by the authors that a neonate diagnosed with CH would adhere to the established protocols for levothyroxine therapy. Therefore, we advocate for physicians to adhere to existing, well-established guidelines. In addition to the reporting deficiency, the overall quality of the evidence was further affected by the generally low methodological quality of the majority of the included studies and the absence of randomized control trials. The rarity of the disorder being studied is the primary reason for the challenge in locating higher-quality studies. Consequently, systematic reviews relying on case series and case reports constitute the sole existing evidence for guideline development. These factors, combined, have led to a diminished quality of available evidence for this study.

In conclusion, this review serves as a foundational resource for devising optimal treatment and care strategies for patients following the early detection and diagnosis o*f NKX2-1*-RD. Furthermore, the cases of these patients illustrate the importance of continuous endocrinological monitoring in individuals with *NKX2-1*-RD, as these alterations manifest in childhood and persist into adulthood. Endocrinologists should be vigilant in identifying and managing these conditions throughout the patient’s lifespan.

**Key points for the treatment and follow-up of thyroid disorders in patients with *NKX2-1-*RD**

1. **Ensuring timely and accurate neonatal screening and screening during childhood and pregnancy for CH.** It is crucial to enable early diagnosis and prompt initiation of LT4 treatment. It is important to note that this recommendation applies to CH in general and is not specific to *NKX2-1*-RD patients.
2. **Initial assessment for all patients with *NKX2-1*-RD.** Neonatal hypothyroidism screening should be systematically verified, and follow-up by an endocrinology department should be ensured, even in the absence of CH. If a blood analysis is conducted for other reasons and the patient does not have a diagnosis of hypothyroidism, it is recommended to include TSH and fT4 in the blood test.
3. **Implementation of standardized guidelines for LT4 dosing,** incorporating factors such as weight, age, and the severity of hypothyroidism. It is important to note that these considerations are general parameters applicable to CH and are not specific to *NKX2-1*-RD patients, as they are already encompassed in existing guidelines for the treatment of hypothyroidism.
4. **Establishment of long-term follow-up for patients with confirmed hypothyroidism and *NKX2-1-*RD**. This strategy will allow us to monitor regular assessments of TSH and fT4 levels, treatment effects, adjust dosages if needed, and address potential side effects to ensure overall clinical success.
5. **Vigilant monitoring** and management of hypothyroidism and transient hypothyroidism in pregnant women.
6. **Management of additional endocrinological disorders such as pituitary hormone deficiencies in *NKX2-1*-RD patients** involves a well-established therapeutic approach by pediatric endocrinologists or endocrinologists for these specific conditions. It is crucial to note that this approach remains uniform and is not substantially influenced by the genetic background, underscoring the consistent nature of treatment strategies for these disorders, irrespective of whether the underlying defect is an *NKX2-1* pathogenic variant or deletion.
7. **Including essential information related to the diagnosis and management of hypothyroidism in future case reports**. Specifically, age and diagnostic methods used for hypothyroidism should be provided, including the actual values of TSH and fT4 levels along with the reference ranges for the specific medical center. Additionally, details such as the age of onset, initial LT4 dosage, and follow-up information for patients undergoing LT4 treatment should be included. Moreover, considering that *NKX2-1*-RD defects may lead to mild hypoplasia of the gland, it is crucial to emphasize in the case reports the inclusion of thyroid ultrasounds, encompassing measurements of the three dimensions, calculation of each lobe’s volume, and reference to normative values for thyroid volume corresponding to the age of the children under consideration.
8. **Provide patient and caregiver education.** This strategy will allow them to recognize the potential side effects of treatment and the importance of adherence to LT4 therapy to enhance treatment compliance and overall health outcomes.

## Supporting information

S1 Checklist PRISMA checklist. Evidence-based items to report in this systematic review on the treatment and follow-up of endocrine diseases in patients with *NKX2-1-*RD.
(DOCX)

S1 File PRISMA checklist. Evidence-based items to report in this systematic review on the treatment and follow-up of endocrine diseases in patients with *NKX2-1-*RD.
(DOCX)

S2 File Search strategy. Presentation of the detailed search strategy used to identify relevant articles related to the treatment and follow-up of endocrine diseases in patients with *NKX2-1*-RD.
(DOCX)

S3 File List of the included and excluded studies and reasons for exclusion. Final number of studies included and excluded in the systematic review, along with the reasons for excluding certain studies at each stage of the screening process.
(DOCX)

S4 File Basal characteristics of patients with *NKX2-1*-RD. Summary of basal characteristics at the patient level.
(DOCX)

S5 File LT4 treatment strategy of patients with *NKX2-1*-RD. Levothyroxine (LT4) treatment strategy for hypothyroidism at the patient level. This table exclusively presents data for patients who underwent LT4 treatment.
(DOCX)

S6 File Quality assessment of included references. Presentation of the quality assessment of included references for the treatment and follow-up of endocrine diseases in patients with *NKX2-1*-RD. **Table A.** Quality assessment of the case reports and case series included in this study. **Table B.** Quality assessment of the cohort study included in this study.
(DOCX)

S7 File Excluded articles and reasons for exclusión. a. Each excluded study is listed along with the specific reason(s) for exclusion, ensuring clarity on why it was not included in the final analysis. b. All studies included in the review are published. No unpublished studies were identified during the selection process.
(XLSX)
