# Supplementary material for: Systematic review of thyroid function in NKX2-1-related disorders: Treatment and follow-up
Source: PLoS One. 2024 Oct 28;19(10):e0309064. doi: 10.1371/journal.pone.0309064 (PMC11515955; doi:10.1371/journal.pone.0309064)
Supplement: S2 File — Presentation of the detailed search strategy used to identify relevant articles related to the treatment and follow-up of endocrine diseases in patients with NKX2-1-RD. (DOCX) [file pone.0309064.s003.docx]

**S2. Search strategy.**  Presentation of the detailed search strategy used to identify relevant articles related to the treatment and follow-up of endocrine diseases in patients with *NKX2-1*-RD.

***MEDLINE (OVID)***

*What are the best procedures for treatment and follow-up of endocrine diseases in patients with NKX2-1-related disorders?*

***Date****: 06/07/2023*

[***https://www.wolterskluwer.com/en/solutions/ovid/ovid-medline-901***](https://www.wolterskluwer.com/en/solutions/ovid/ovid-medline-901)

| **ID** | **Searches** |
| --- | --- |
| 1 | exp Thyroid Nuclear Factor 1/ or 'thyroid nuclear factor 1*'.ti,ab,kw. or 'thyroid transcription factor*'.ti,ab,kw. or 'thyroid-specific enhancer-binding protein*'.ti,ab,kw. or 't-ebp'.ti,ab,kw. or 'tebp'.ti,ab,kw. or 'titf1'.ti,ab,kw. or 'titf 1'.ti,ab,kw. or 'titf-1'.ti,ab,kw. or 'ttf1'.ti,ab,kw. or 'ttf 1'.ti,ab,kw. or 'ttf-1'.ti,ab,kw. |
| 2 | ('nkx2 1*' or 'nkx2-1*' or 'nkx2?1*' or 'nkx 2 1*' or 'nkx 2-1*' or 'nkx 2?1*' or 'nk2 homeobox 1' or 'nkx2 homeodomain transcription*' or 'nk-2 homolog a*' or 'nkx2a*').ti,ab,kw. |
| 3 | PAX9 Transcription Factor/ or 'pax9*'.ti,ab,kw. or 'pax-9*'.ti,ab,kw. or 'pax 9*'.ti,ab,kw. or 'pair box 9*'.ti,ab,kw. or 14q12*.ti,ab,kw. or 14q13*.ti,ab,kw. |
| 4 | ((hereditar* adj3 chorea*) or (benign* adj3 chorea*) or 'brain lung thyroid*' or 'brain-lung-thyroid' or 'brain-thyroid-lung' or 'brain thyroid lung').ti,ab,kw. |
| 5 | 1 or 2 or 3 or 4 |
| 6 | exp Congenital Hypothyroidism/ or hypothyroidism*.ti,ab,kw. |
| 7 | Thyroid Diseases/ge or exp Thyroid Dysgenesis/ge or (thyroid dysgenesis or dyshormonogenesis*).ti,ab,kw. |
| 8 | exp Abnormalities, Multiple/ or pedigree.ti,ab,kw. or familial.ti,ab,kw. or linkage.ti,ab,kw. or kindred.ti,ab,kw. |
| 9 | 6 or 7 or 8 |
| 10 | 5 and 9 |
| 11 | exp Therapeutics/ or followup.ti,ab,kw. or 'follow up'.ti,ab,kw. or monitor*.ti,ab,kw. or therap*.ti,ab,kw. or treat*.ti,ab,kw. |
| 12 | (Levothyroxin* or L-T4 or LT4 or tiche or soloxine).ti,ab,kw. |
| 13 | ((Thyrotropin* or Thyroid hormone* or TH or thyrotropin or TSH or thyroid stimulating hormone or free thyroxine or fT4) adj5 (test* or level* or concentration or measur* or threshold* or analys*)).ti,ab,kw. |
| 14 | exp Ultrasonography/ or (ultrasound or ultrasonog* or US or sonograp* or examination).ti,ab,kw. |
| 15 | exp Hearing tests/ |
| 16 | 11 or 12 or 13 or 14 or 15 |
| **17** | **10 and 16** |
