# Supplementary material for: Systematic review of thyroid function in NKX2-1-related disorders: Treatment and follow-up
Source: PLoS One. 2024 Oct 28;19(10):e0309064. doi: 10.1371/journal.pone.0309064 (PMC11515955; doi:10.1371/journal.pone.0309064)
Supplement: S3 File — Final number of studies included and excluded in the systematic review, along with the reasons for excluding certain studies at each stage of the screening process. (DOCX) [file pone.0309064.s004.docx]

**S3. List of the included and excluded studies and reasons for exclusion.** Final number of studies included and excluded in the systematic review, along with the reasons for excluding certain studies at each stage of the screening process.

| **INCLUDED STUDIES** |
| --- |
| 1. Asmus F, Horber V, Pohlenz J, Schwabe D, Zimprich A, Munz M, et al. A novel TITF-1 mutation causes benign hereditary chorea with response to levodopa. Neurology. 2005;64(11):1952-4. 2. Balicza P, Grosz Z, Molnár V, Illés A, Csabán D, Gézsi A, et al. NKX2-1 New Mutation Associated With Myoclonus, Dystonia, and Pituitary Involvement. Front Genet. 2018;9:335. |
| 1. Barnett CP, Mencel JJ, Gecz J, Waters W, Kirwin SM, Vinette KM, et al. Choreoathetosis, congenital hypothyroidism and neonatal respiratory distress syndrome with intact NKX2-1. Am J Med Genet A. 2012;158a(12):3168-73. |
| 1. Barreiro J, Alonso-Fernández JR, Castro-Feijoo L, Colón C, Cabanas P, Heredia C, et al. Congenital hypothyroidism with neurological and respiratory alterations: a case detected using a variable diagnostic threshold for TSH. J Clin Res Pediatr Endocrinol. 2011;3(4):208-11. |
| 1. Carré A, Szinnai G, Castanet M, Sura-Trueba S, Tron E, Broutin-L'Hermite I, et al. Five new TTF1/NKX2.1 mutations in brain-lung-thyroid syndrome: rescue by PAX8 synergism in one case. Hum Mol Genet. 2009;18(12):2266-76. |
| 1. Delestrain C, Aissat A, Nattes E, Gibertini I, Lacroze V, Simon S, et al. Deciphering an isolated lung phenotype of NKX2-1 frameshift pathogenic variant. Front Pediatr. 2022;10:978598. |
| 1. Doyle DA, Gonzalez I, Thomas B, Scavina M. Autosomal dominant transmission of congenital hypothyroidism, neonatal respiratory distress, and ataxia caused by a mutation of NKX2-1. J Pediatr. 2004;145(2):190-3. |
| 1. Ferrara JM, Adam OR, Kirwin SM, Houghton DJ, Shepherd C, Vinette KM, et al. Brain-lung-thyroid disease: clinical features of a kindred with a novel thyroid transcription factor 1 mutation. J Child Neurol. 2012;27(1):68-73. |
| 1. Fons C, Rizzu P, Garcia-Cazorla A, Martorell L, Ormazabal A, Artuch R, et al. TITF-1 gene mutation in a case of sporadic non-progressive chorea. Response to levodopa treatment. Brain Dev. 2012;34(3):255-7. 2. Galambos C, Levy H, Cannon CL, Vargas SO, Reid LM, Cleveland R, et al. Pulmonary pathology in thyroid transcription factor-1 deficiency syndrome. Am J Respir Crit Care Med. 2010;182(4):549-54. 3. Gonçalves D, Lourenço L, Guardiano M, Castro-Correia C, Sampaio M, Leão M. Chiari Malformation Type I in a Patient with a Novel NKX2-1 Mutation. J Pediatr Neurosci. 2019;14(3):169-72. |
| 1. Gras D, Jonard L, Roze E, Chantot-Bastaraud S, Koht J, Motte J, et al. Benign hereditary chorea: phenotype, prognosis, therapeutic outcome and long term follow-up in a large series with new mutations in the TITF1/NKX2-1 gene. J Neurol Neurosurg Psychiatry. 2012;83(10):956-62. 2. Gu R, Ye G, Zhou Y, Jiang Z. Combined mutations of NKX2-1 and surfactant protein C genes for refractory low oxyhemoglobin saturation and interstitial pneumonia: A case report. Medicine (Baltimore). 2020;99(12):e19650. |
| 1. Hayasaka I, Cho K, Akimoto T, Ikeda M, Uzuki Y, Yamada M, et al. Genetic basis for childhood interstitial lung disease among Japanese infants and children. Pediatr Res. 2018;83(2):477-83. |
| 1. Kharbanda M, Hermanns P, Jones J, Pohlenz J, Horrocks I, Donaldson M. A further case of brain-lung-thyroid syndrome with deletion proximal to NKX2-1. Eur J Med Genet. 2017;60(5):257-60. |
| 1. Kleinlein B, Griese M, Liebisch G, Krude H, Lohse P, Aslanidis C, et al. Fatal neonatal respiratory failure in an infant with congenital hypothyroidism due to haploinsufficiency of the NKX2-1 gene: alteration of pulmonary surfactant homeostasis. Arch Dis Child Fetal Neonatal Ed. 2011;96(6):F453-6. |
| 1. Koht J, Løstegaard SO, Wedding I, Vidailhet M, Louha M, Tallaksen CM. Benign hereditary chorea, not only chorea: a family case presentation. Cerebellum Ataxias. 2016;3:3. |
| 1. Krude H, Schütz B, Biebermann H, von Moers A, Schnabel D, Neitzel H, et al. Choreoathetosis, hypothyroidism, and pulmonary alterations due to human NKX2-1 haploinsufficiency. J Clin Invest. 2002;109(4):475-80. 2. Kumar G, Dixon A. Benign hereditary chorea: a case report and brief review of inherited choreas. Pediatr Neurol. 2014;51(4):532-6. |
| 1. Li M, Li Z, Chen M, Hu Z, Zhou M, Wu L, et al. Novel Missense Variants in PAX8 and NKX2-1 Cause Congenital Hypothyroidism. Int J Mol Sci. 2023;24(1). |
| 1. Lynn MM, Simon D, Kasi AS. Hypoxaemia and interstitial lung disease in an infant with hypothyroidism and hypotonia. BMJ Case Rep. 2020;13(12). |
| 1. Makretskaya N, Bezlepkina O, Kolodkina A, Kiyaev A, Vasilyev EV, Petrov V, et al. High frequency of mutations in 'dyshormonogenesis genes' in severe congenital hypothyroidism. PLoS One. 2018;13(9):e0204323. 2. Maric N LO. Pulmonary Hemorrhage in a Patient with Brain-Lung-Thyroid Syndrome Caused by a p.T86fs Variant in the NKX2-1 Gene. . Central Eur J Paed. 2020;16(2):155-160. |
| 1. Moya CM, Zaballos MA, Garzón L, Luna C, Simón R, Yaffe MB, et al. TAZ/WWTR1 Mediates the Pulmonary Effects of NKX2-1 Mutations in Brain-Lung-Thyroid Syndrome. J Clin Endocrinol Metab. 2018;103(3):839-52. 2. Nagasaki K, Narumi S, Asami T, Kikuchi T, Hasegawa T, Uchiyama M. Mutation of a gene for thyroid transcription factor-1 (TITF1) in a patient with clinical features of resistance to thyrotropin. Endocr J. 2008;55(5):875-8. |
| 1. Nattes E, Lejeune S, Carsin A, Borie R, Gibertini I, Balinotti J, et al. Heterogeneity of lung disease associated with NK2 homeobox 1 mutations. Respir Med. 2017;129:16-23. |
| 1. Parnes M, Bashir H, Jankovic J. Is Benign Hereditary Chorea Really Benign? Brain-Lung-Thyroid Syndrome Caused by NKX2-1 Mutations. Mov Disord Clin Pract. 2019;6(1):34-9. |
| 1. Prasad R, Nicholas AK, Schoenmakers N, Barton J. Haploinsufficiency of NKX2-1 in Brain-Lung-Thyroid Syndrome with Additional Multiple Pituitary Dysfunction. Horm Res Paediatr. 2019;92(5):340-4. 2. Provenzano C, Zamboni M, Veneziano L, Mantuano E, Garavaglia B, Zorzi G, et al. Functional characterization of two novel mutations in TTF-1/NKX2.1 homeodomain in patients with benign hereditary chorea. J Neurol Sci. 2016;360:78-83. 3. Safi KH, Bernat JA, Keegan CE, Ahmad A, Hershenson MB, Arteta M. Interstitial lung disease of infancy caused by a new NKX2-1 mutation. Clin Case Rep. 2017;5(6):739-43. |
| 1. Salerno T, Peca D, Menchini L, Schiavino A, Petreschi F, Occasi F, et al. Respiratory insufficiency in a newborn with congenital hypothyroidism due to a new mutation of TTF-1/NKX2.1 gene. Pediatr Pulmonol. 2014;49(3):E42-4. |
| 1. Salvado M, Boronat-Guerrero S, Hernández-Vara J, Álvarez-Sabin J. [Chorea due to TITF1/NKX2-1 mutation: phenotypical description and therapeutic response in a family]. Rev Neurol. 2013;56(10):515-20. |
| 1. Salvatore E, Di Maio L, Filla A, Ferrara AM, Rinaldi C, Saccà F, et al. Benign hereditary chorea: clinical and neuroimaging features in an Italian family. Mov Disord. 2010;25(10):1491-6. |
| 1. Santos-Silva R, Rosário M, Grangeia A, Costa C, Castro-Correia C, Alonso I, et al. Genetic analyses in a cohort of Portuguese pediatric patients with congenital hypothyroidism. J Pediatr Endocrinol Metab. 2019;32(11):1265-73. |
| 1. Shiohama T, Ohashi H, Shimizu K, Fujii K, Oba D, Takatani T, et al. l-Thyroxine-responsive drop attacks in childhood benign hereditary chorea: A case report. Brain Dev. 2018;40(4):353-6. |
| 1. Tanaka T, Aoyama K, Suzuki A, Saitoh S, Mizuno H. Clinical and genetic investigation of 136 Japanese patients with congenital hypothyroidism. J Pediatr Endocrinol Metab. 2020;33(6):691-701. 2. Thust S, Veneziano L, Parkinson MH, Bhatia KP, Mantuano E, Gonzalez-Robles C, et al. Altered pituitary morphology as a sign of benign hereditary chorea caused by TITF1/NKX2.1 mutations. Neurogenetics. 2022;23(2):91-102. |
| 1. Trevisani V, Predieri B, Madeo SF, Fusco C, Garavelli L, Caraffi S, et al. Growth hormone deficiency in a child with benign hereditary chorea caused by a de novo mutation of the TITF1/NKX2-1 gene. J Pediatr Endocrinol Metab. 2022;35(3):411-5. |
| 1. Uematsu M, Haginoya K, Kikuchi A, Nakayama T, Kakisaka Y, Numata Y, et al. Hypoperfusion in caudate nuclei in patients with brain-lung-thyroid syndrome. J Neurol Sci. 2012;315(1-2):77-81. |
| 1. Veneziano L, Parkinson MH, Mantuano E, Frontali M, Bhatia KP, Giunti P. A novel de novo mutation of the TITF1/NKX2-1 gene causing ataxia, benign hereditary chorea, hypothyroidism and a pituitary mass in a UK family and review of the literature. Cerebellum. 2014;13(5):588-95. |
| 1. Villafuerte B, Natera-de-Benito D, González A, Mori MA, Palomares M, Nevado J, et al. The Brain-Lung-Thyroid syndrome (BLTS): A novel deletion in chromosome 14q13.2-q21.1 expands the phenotype to humoral immunodeficiency. Eur J Med Genet. 2018;61(7):393-8. |
| 1. Villamil-Osorio M, Yunis LK, Quintero L, Restrepo-Gualteros S, Yunis JJ, Jaramillo L, et al. [Brain-lung-thyroid syndrome in a newborn with deletion 14q12-q21.1]. Andes Pediatr. 2021;92(6):930-6. |

**EXCLUDED STUDIES**

| **Wrong patient population** |
| --- |
| 1. Costa MC, Costa C, Silva AP, Evangelista P, Santos L, Ferro A, Sequeiros J, Maciel P. Nonsense mutation in TITF1 in a Portuguese family with benign hereditary chorea. Neurogenetics. 2005 Dec;6(4):209-15. 2. Konishi T, Kono S, Fujimoto M, Terada T, Matsushita K, Ouchi Y, Miyajima H. Benign hereditary chorea: dopaminergic brain imaging in patients with a novel intronic NKX2.1 gene mutation. J Neurol. 2013 Jan;260(1):207-13. 3. Li L, Li X, Wang X, Han M, Zhao D, Wang F, Liu S. Mutation screening of eight genes and comparison of the clinical data in a Chinese cohort with congenital hypothyroidism. Endocrine. 2023 Jan;79(1):125-134. 4. McMichael G, Haan E, Gardner A, Yap TY, Thompson S, Ouvrier R, Dale RC, Gecz J, Maclennan AH. NKX2-1 mutation in a family diagnosed with ataxic dyskinetic cerebral palsy. Eur J Med Genet. 2013 Sep;56(9):506-9. 5. Rosati A, Berti B, Melani F, Cellini E, Procopio E, Guerrini R. Recurrent drop attacks in early childhood as presenting symptom of benign hereditary chorea caused by TITF1 gene mutations. Dev Med Child Neurol. 2015 Aug;57(8):777-9. 6. Thomann J, Tittel SR, Voss E, Oeverink R, Palm K, Fricke-Otto S, Kapelari K, Holl RW, Woelfle J, Bettendorf M. Guideline Adherence and Registry Recruitment of Congenital Primary Hypothyroidism: Data from the German Registry for Congenital Hypothyroidism (HypoDok). Int J Neonatal Screen. 2021 Feb 12;7(1):10. 7. Ye L, Yin Y, Chen M, Gong N, Peng Y, Liu H, Miao J. Combined genetic screening and traditional newborn screening to improve the screening efficiency of congenital hypothyroidism. Front Pediatr. 2023 May 12;11:1185802. 8. Yu B, Long W, Yang Y, Wang Y, Jiang L, Cai Z, Wang H. Newborn Screening and Molecular Profile of Congenital Hypothyroidism in a Chinese Population. Front Genet. 2018 Oct 29;9:509. 9. Yuan ZF, Mao HQ, Luo YF, Wu YD, Shen Z, Zhao ZY. Thyrotropin receptor and thyroid transcription factor-1 genes variant in Chinese children with congenital hypothyroidism. Endocr J. 2008 May;55(2):415-23. |
| **Wrong outcomes** |
| 1. Hamvas A, Deterding RR, Wert SE, White FV, Dishop MK, Alfano DN, Halbower AC, Planer B, Stephan MJ, Uchida DA, Williames LD, Rosenfeld JA, Lebel RR, Young LR, Cole FS, Nogee LM. Heterogeneous pulmonary phenotypes associated with mutations in the thyroid transcription factor gene NKX2-1. Chest. 2013 Sep;144(3):794-804. 2. LeMoine BD, Browne LP, Liptzin DR, Deterding RR, Galambos C, Weinman JP. High-resolution computed tomography findings of thyroid transcription factor 1 deficiency (NKX2-1 mutations). Pediatr Radiol. 2019 Jun;49(7):869-875. 3. Thorwarth A, Schnittert-Hübener S, Schrumpf P, Müller I, Jyrch S, Dame C, Biebermann H, Kleinau G, Katchanov J, Schuelke M, Ebert G, Steininger A, Bönnemann C, Brockmann K, Christen HJ, Crock P, deZegher F, Griese M, Hewitt J, Ivarsson S, Hübner C, Kapelari K, Plecko B, Rating D, Stoeva I, Ropers HH, Grüters A, Ullmann R, Krude H. Comprehensive genotyping and clinical characterisation reveal 27 novel NKX2-1 mutations and expand the phenotypic spectrum. J Med Genet. 2014 Jun;51(6):375-87. 4. Young LR, Deutsch GH, Bokulic RE, Brody AS, Nogee LM. A mutation in TTF1/NKX2.1 is associated with familial neuroendocrine cell hyperplasia of infancy. Chest. 2013 Oct;144(4):1199-1206. |

**Wrong publication type**

| 1. Akkari M, Ben Rhouma H, Klaa H, Rouissi A, Kraoua I, Turki I. Benign hereditary chorea: A Tunisian family case presentation. Mov. Disord. 2019;34 (Tunis, Tunisia):S123. 2. Auyeung V, Planer BC, Chartoff A, Oundjian N. The link between respiratory failure and congenital hypothyroidism. Center for Children, Joseph M. Sanzari Children's Hospital, Hackensack University Medical Center, Hackensack, NJ, United States): Thyroid. 2010. A52-A53. 3. Baldan F, Cavaliere E, Gortan AJ, Passon N, Fabbro D, Marin D, Carecchio M, Credendino SC, Gallo R, Cogo P, Damante G, De Vita G. A case of familial brain-lung-thyroid syndrome due to a NKX2.1 run-on mutation. European Journal of Human Genetics 2022;30(SUPPL 1):150. 4. Borie R, Kannengiesser C, Amselem S, Brugiere O, Bouvry D, Clement A. Cottin V, Dieude P, Dupin C, Epaud R, Gouya L, Fanen P, De Fontbrune FS, Wemeau-Stervinou L, Nathan N, Crestani B. Multidisciplinary team dedicated to suspected heritable pulmonary fibrosis. Eur. Respir. J. 2018;52(Hopital Bichat, Paris, France). 5. Burglen L, Ravelli C, Louha M, Qebibo L, Afenjar A, Mignot C, Rodriguez D, Doummar D. Childhood onset chorea: an overview of genetic etiologies in a series of 85 patients. European Journal of Human Genetics 2023;31,197. 6. Coon EA, Milone M, Niu Z, Patterson MC, Ahlskog JE. Neurologic and systemic variability in benign hereditary chorea due to NKX2-1 mutations. Mov. Disord. 2015;30(Rochester, MN, United States):S139. 7. De Gusmao CM, Kok F, Casella EB, Waugh JL. Benign hereditary chorea related to NKX2-1 with ataxia and dystonia. Neurol. Genet. 2016;2(1). 8. Deshpande A, Dutta S, Singha A, Mukhopadhyay P, Ghosh S. Abstract 125: Congenital hypothyroidism: Clinical profile and genetic abnormalities. Indian Journal of Endocrinology and Metabolism. 2022. 26(Suppl 8):p S53-S54. 9. Deterding RR, Dishop M, Uchida DA, Stephan M, Williames L, Lebel RR, Halbower AC, Rosenfeld J, Moffitt D, Wert SE, Nogee L. Thyroid transcription factor 1 gene abnormalities: An under recognized cause of children's interstitial lung disease. Am. J. Respir. Crit. Care Med. 2010;181(1). 10. Du Souich C, Scocchia A, Gall K, Hathaway J, Taylor A, Huusko J, Bernal M, Saarinen I, Schleit J, Paananen J, Myllykangas S, Koskenvuo J. Characterization of molecular diagnostic findings in an unselected cohort with suspected congenital hypothyroidism or resistance to thyroid hormone. European Journal of Human Genetics 2023;31():120. 11. El Taoum K, Com G, Warren, R. An infant with congenital hypothyroidism and labored breathing: A case report. Am. J. Respir. Crit. Care Med. 2014;189 (University of Arkansas for Medical Sciences, Little Rock, AR, United States). 12. Ferrara, JM, Adam OR, Kirwin SM, Houghton, DJ, Litvan I. Brain-lung-thyroid disease, (BLT) - Clinical features of a kindred with a novel TITF-1 mutation Mov. Disord. 2011;26 (Louisville, KY, United States):S33-S34. 13. Ferreira SH, Pereia S, Jacob S, Abreu M, Gonçalves D, Sampaio M, Leão M, Castro-Correia C, Fontoura, M. Congenital hypothyroidism and Brain-Lung-Thyroid syndrome. Cogent Med. 2017;4(1). 14. Gonzalez A, Villafuerte B, Jimenez P, Arguinzoniz L, De La Luz Ruiz M, Villarroel C, Calzada R, Robles C, Moreno JC. Brain-lung-thyroid syndrome (BLTS) in a mexican patient with a novel intragenic deletion in NKX2-1. Horm. Res. Paediatr. 2017;88 (National Institute of Pediatrics INP, Mexico City, Mexico):592. 15. Graf S, Bösch N, Bachmann S, Zumsteg U, Heinimann K, Szinnai G. Familial brain-lung-thyroid syndrome due to a new NKX2-1 mutation p.Q172L causing disabling benign hereditary chorea. Horm. Res. Paediatr. 2012;78 (University Children's Hospital Basel, Paediatric Endocrinology, Basel, Switzerland):127. 16. Guala A, Pinna N, Malinverni A, Nocita G, Heutink P, Spadoni E, Danesino C. Benign Hereditary Chorea: From clinic to basic research... and back. Ital. J. Pediatr. 2003;29(4):257. 17. Haubenberger D, Bauer P, Lieba-Samal D, Zimprich A, Auff E, Pirker W. A novel NKX2A-mutation causing benign hereditary chorea is associated with non-progressive striatal D2-receptor dysfunction. Mov. Disord. 2012;27 (Vienna, Austria):S327. 18. Hermanns P, Kumorowicz-Czoch M, Pohlenz J. Mutations in the TTF1 and the PAX8 genes in a boy with thyroid dysgenesis, respiratory and neurological disorders. Horm. Res. Paediatr. 2013;80 (Johannes Gutenberg University Medical School, Department of Pediatrics, Mainz, Germany):194. 19. Hicks J, Mierau G, Wartchow E, Langston C. TTF-1 associated surfactant deficiency: Role of ultrastructural evaluation. Lab. Invest. 2013;93 (Baylor College of Medicine and Texas Children's Hospital, Houston, TX, United States of America; Children's Hospital Colorado, Aurora, CO, United States of America):504A. 20. Jung SY, Lee J. Analysis of hypothyroidism NGS test in Korean patients with congenital hypothyroidism in a single center. Horm. Res. Paediatr.2021; 94(SUPPL 1):172-173. 21. Keskin M, Yalcin A, Oztuzcu S, Kor Y, Coskun Y. The role of PAX-8, TTF-1 and TTF-2 gene polymorphism in children with congenital hypothyroidism due to thyroid dysgenesis. Horm. Res. Paediatr. 2012;78(Gaziantep University, Faculty of Medicine, Pediatric Endocrinology and Metabolism, Gaziantep, Turkey):241-242. 22. Levaillant L, Bouhours-Nouet N, Illouz F, Bouzamondo N, Rodien P, Prunier-Mirebeau D, Coutant R. Genetic analyses in patients having congenital hypothyroidism with gland-in-situ by nextgeneration sequencing. Horm. Res. Paediatr. 2021;94(SUPPL 1):61. 23. Lourenço L, Reis E Melo A, Gonçalves D, Sampaio M, Correia C, Guardiano M. Coreoathetosis and congenital hypothyroidism. Cogent Med. 2017;4(1). 24. Lumsden DE, Turnbull J, Josifov DJ, Dlamini N, Jungbluth H. Choreoathetoid movement disorder due to a 14q13 deletion including the NKX2-1 gene encoding thyroid transcription factor-1 (TITF1). Dev. Med. Child Neurol. 2012;54 (Paediatric Neurology, Evelina Children's Hospital, London, United Kingdom):16. 25. MacLean J, Luc Q, Ramos-Platt L, Saitta S, Quindipan C. NKX2-1-related disorder with cerebral folate deficiency. Mov. Disord. 2019;34 (Los Angeles, CA, United States):S216. 26. Maristella S, Deborah B, Gabor S, Karl H, Britta S, Maya S, Emma FC. Brain-lung-thyroid syndrome due to a new NKX2-1 mutation. Swiss Med. Wkly 2019;149 (Department of Pediatric Endocrinology, Diabetology and Metabolism, Inselspital, Bern University Children's Hospital, Switzerland):22S. 27. Mook A, Kilbane C. Benign hereditary chorea with nocturnal dyskinesias. Mov. Disord. 2018;33(Cleveland, OH, United States):S311. 28. Morandini M, Leger J, Polak M, Castanet M, Congenital H. Psychomotor delay in patients with congenital hypothyroidism: Inadequate treatment or genetic syndrome?. Horm. Res. Paediatr. 2010;74 (Université Paris Descartes, Pediatric Endocrinology Unit, Paris, France):88-89. 29. Nathan N, Borie R, Jovien S, Doummar D, Louha M, Beucher J, Henriat M, Breton E, Clement A. Phenotype heterogeneity in a familial “brain lung thyroid syndrome” related to a novel NKX-2.1 mutation. Eur. Respir. J. 2016;48. 30. Ni J, Cameron J, Hopper RK, Tan S, Si X. A Rare Case of Interstitial Lung Disease and Pulmonary Hypertension Associated With NKX2- 1 Mutation. American Journal of Respiratory and Critical Care Medicine 2023;207(1). 31. Park J, Kim, S, Lee J. Next Generation Sequencing Analysis of Congenital Hypothyroidism Patients in A Single Tertiary Center. Hormone Research in Paediatrics 2022;95,412. 32. Patianna VD, Predieri B, Garavelli L, Fusco C, Madeo SF, Bruzzi P, Iughetti L. A novel mutation in the TITF1 gene in a child with benign hereditary chorea. Horm. Res. Paediatr. 2014;82 (Department of Medical and Surgical Sciences Mother, Children and Adult, University of Modena and Reggio Emilia, Modena, Italy):167. |
| --- |
| 1. Peake D, Cassidy D, Mckee S, King MD, Kuria MA. A novel TITF1 mutation in benign hereditary chorea. Dev. Med. Child Neurol. 2012;54 (Paediatric Neurology, Royal Belfast Hospital for Sick Children, Belfast, United Kingdom):82. 2. Peall KJ, Lumsden D, Morris HR, Jungbluth H, Kurian MA. NKX2-1 mutations in brain-lung-thyroid syndrome: A case series. Dev. Med. Child Neurol. 2013;55 (Neurology, University Hospital of Wales, Cardiff, United Kingdom). 3. Reem I, Jacqueline S, Bishay Lara C. PULMONARY HYPERTENSION in A LIMPING CHILD: A CASE of URGEN-C. Pulmonary Circulation 2022;12(2). 4. Sempere AP, Aparicio SMS, Pérez-Tur J. Benign hereditary chorea: Clinical features and long-term follow-up in a Spanish family. Parkinsonism & Related Disorders 2013;19(3):394-396. 5. Shukla V, Samanta D, Habetz K. Levodopa responsive chorea with TITFi mutation in brain-lung-thyroid disease. Neurology 2017;88(16). 6. Vigone MC, Saracco L, Vincenzi G, Caiulo S, Di Frenna M, Persani L, De Filippis T, Guizzardi F, Patricelli MG, Spiga I, Weber G. The genetic and clinical characteristic of pediatric patients with congenital hypothyroidism gland in-situ. Horm. Res. Paediatr. 2019;91(Ospedale San Raffaele, Milan, Italy):338 7. Villafuerte B, De Benito DN, Lacamara N, Garcia M, Lumbreras C, De Randamie R, Nevado J, Moreno, JC. Identification of a “Cryptic” de novo deletion in NKX2.1 in the Brain-Lung-Thyroid syndrome using genomic SNP arrays. Horm. Res. Paediatr. 2016;86 (Molecular Laboratory, Institute for Medical and Molecular Genetics (INGEMM), La Paz University Hospital, Madrid, Spain):491. 8. Wouters L, De Bruyn G, De Waele , Jansen K, Francois I, Reynaert N, Buyse G, Lagae L, Goemans N. The brain-lung-thyroid syndrome as a rare cause of chorea: Case report of a novel mutation in the thyroid transcription factor-1 (TITF-1) gene. Eur. J. Paediatr. Neurol. 2013;17 (UZ Leuven, Belgium):S104. |
| 1. Yang J, Chung WY, Oh SH, Seo GH, Kim JR, Yu J. A case of permanent congenital hypothyroidism with NKX2-1 mutation and optic nerve thickness. Hormone Research in Paediatrics 2022;95():587. |
| 1. Yilmaz AA, Erdeve SS, Yuksel D, Oztoprak U, Cetinkaya S. A rare cause of congenital hypothyroidism: Brain-lung-thyroid syndrome. Horm. Res. Paediatr. 2021;94(SUPPL 1):413-414. |
| **Wrong language** |
| 1. Liang R, Ou S, Ding Y, Liu C. A case of brain**-**lung**-**thyroid syndrome. Zhong Nan Da Xue Xue Bao Yi Xue Ban. 2022 Mar 28;47(3):396-400. English, Chinese. |

| **Non-available text** |
| --- |
| 1. Heidari MM, Madani Manshadi SA, Eshghi AR, Talebi F, Khatami M, Bragança J, Ordooei M, Chamani R, Ghasemi F. Mutational and bioinformatics analysis of the NKX2.1 gene in a cohort of Iranian pediatric patients with congenital hypothyroidism (CH). Physiol Int. 2022 Jun 7;109(2):261-277. 2. Kojima Y, Atobe M, Aoki Y, Suzuki M, Itomi K, Tanaka T, Saitoh S. A case of brain-lung-thyroid syndrome showing atypical symptoms. No To Hattatsu 2021;53(1):44-48 3. Peters B, Van Mossevelde PWJ, Van Trotsenburg ASP. Neonatal respiratory distress of unknown origin: Do not forget the thyroid gland. Tijdschr. Geneeskd. 2017;73(5):289-294. |
