# Supplementary material for: Systematic review of thyroid function in NKX2-1-related disorders: Treatment and follow-up
Source: PLoS One. 2024 Oct 28;19(10):e0309064. doi: 10.1371/journal.pone.0309064 (PMC11515955; doi:10.1371/journal.pone.0309064)
Supplement: S4 File — Summary of basal characteristics at the patient level. (DOCX) [file pone.0309064.s005.docx]

**S4. Basal characteristics of patients with *NKX2-1*-RD.** Summary of basal characteristics at the patient level.

| **Patient and reference** | **Sex^a^** | **Age^b^** | **NKX2-1 mutation (gene test^c^)** | **Inherited^d^** | **Endocrine alterations^e^** | **Chorea, movement disorders** | **Respiratory affections** | **Other clinical data** |
| --- | --- | --- | --- | --- | --- | --- | --- | --- |
| **Asmus_2005_PII:3** | F | NA | Nonsense, *NKX2-1*(NM_003317.4):c.523G>T/  p.Glu175* (sequencing) | D | CH | chorea, gait disorder. | NA | NA |
| **Asmus_2005_PIII:1** | F | NA | Nonsense, *NKX2-1*(NM_003317.4):c.523G>T/  p.Glu175* (sequencing) | M | CH | gait disorder, muscular hypotonia. | Neonatal respiratory distress, pneumonia. | NA |
| **Asmus_2005_PIII:2** | M | 8 y | Nonsense, *NKX2-1*(NM_003317.4):c.523G>T/  p.Glu175* (sequencing) | M | CH | chorea, gait disorder, muscular hypotonia, dysarthria. | Neonatal respiratory distress, pneumonia. | The patient passed away at 8 years (lymphocytic leukemia). |
| **Asmus_2005_PIII:4** | F | NA | Nonsense, *NKX2-1*(NM_003317.4):c.523G>T/  p.Glu175* (sequencing) | M | CH | chorea, gait disorder, muscular hypotonia. | Neonatal respiratory distress, pneumonia. | NA |
| **Balicza_2018_PII/2** | M | 46 y | Frameshift, *NKX2-1* (NM_003317):  c.338G>A, p.Trp113*  (sequencing) | NA | H, testosterone and luteinizing hormone deficiency, hypogonadism, pituitary disfunction. | Chorea, ataxia, dyskinesia, hyperkinesia, dystonia, dysarthria, developmental delay. | Asthma. | Hepatic steatosis. |
| **Barnett_2012_P1** | F | 1 y 11 m | Deletion 14q13.1-q13.3 (CGH array) (CGH array) | D | CH, hypoplastic pituitary gland. | Chorea, ataxia, muscular hypotonia, hyperkinesia. | Neonatal respiratory distress, respiratory infections, chronic cough. | Developmental delay, esotropia, facial dysmorphism. |
| **Barreiro_2011_P1** | M | 3 y | Splicing, *NKX2-1* (ENST00000354822.7):c.464-1G>A (NA) | NA | CH | Chorea, mild hypotonia, dystonia, motor retardation. | Neonatal respiratory distress, bronchial hyperactivity, bronchiolitis. | Removal of tonsils and adenoids. |
| **Carré_2009_P1** | F | 6 y | Deletion 14q13 (FISH) | D | Mild CH (CpH later) | Chorea, ataxia, muscular hypotonia | Neonatal respiratory distress, pulmonary infections | Developmental delay, corpus callosum agenesis. |
| **Carré_2009_P2TA** | NA | 16 y | Splicing, *NKX2-1* (ENST00000354822.7):c.464-1G>A (sequencing) | D | CH | Chorea, muscular hypotonia. | Neonatal respiratory distress, pulmonary infections | Developmental delay. |
| **Carré_2009_P3TB** | NA | 15 y | Splicing, NKX2-1, (NM_?), c.376-2A>G/p.? (sequencing) | D | CH | Chorea | Neonatal respiratory distress, | NA |
| **Carré_2009_P4** | M | 14 y | Missense, *NKX2-1* (NM_003317.4):  c.526C>G/  p.Leu176Val (sequencing) | D | H | Chorea, muscular hypotonia | bronchitis | Developmental delay. |
| **Carré_2009_P5** | F | 1 y 6 m | Missense, NKX2-1 (NM_003317.4):  c.605C>T/  p.Pro202Leu (sequencing) | D | CH | Chorea, muscular hypotonia. | NA | Developmental delay. |
| **Carré_2009_P6** | F | NA | Missense, *NKX2-1* (NM_003317.4):  c.629A>C/  p.Gln210Pro (sequencing) | D | CH | Chorea, muscular hypotonia. | NA | Developmental delay. |
| **Delestrain_2023_P2** | M | NA | Frameshift, *NKX2-1* (NM_003317.4):  c.254dup/  p.Tyr86LeufsTer323 (sequencing) | D | CH | Chorea, hypotonia, motor delay, chorea. | Neonatal respiratory distress, hyaline membrane disease | NA |
| **Doyle_2004_P I1** | F | 59 y | Missense, *NKX2-1* (NM_001079668.3),c.740A>G/p.Lys247Arg (sequencing) | NA | H | Muscular hypotonia | pneumonia | Stammering |
| **Doyle_2004_P II2** | F | NA | Missense, *NKX2-1* (NM_001079668.3),c.740A>G/p.Lys247Arg (sequencing) | M | H | Cerebral palsy, | NA | Stammering, constipation |
| **Doyle_2004_P III2** | M | 10 y | Missense, *NKX2-1* (NM_001079668.3),c.740A>G/p.Lys247Arg (sequencing) | M | CH | Chorea, ataxia, dysarthria | Neonatal respiratory distress | Developmental delay. |
| **Doyle_2004_P III3** | F | 4 y | Missense, *NKX2-1* (NM_001079668.3),c.740A>G/p.Lys247Arg (sequencing) | M | CH | Chorea, ataxia, dysarthria | Neonatal respiratory distress | Developmental delay. |
| **Ferrara_2012_PII-2 (proband)** | M | 31 y | Missense, *NKX2-1*(ENST00000354822.7):c.617T>A  p.Leu206Gln (sequencing) | M | SH | Chorea, dystonia, dyspnea, ataxia, limb weakness, dysarthria. | NA | Learning difficulties, epilepsy. |
| **Ferrara_2012_PIII-2** | F | 2 y 6 m | Missense, *NKX2-1*(ENST00000354822.7):c.617T>A  p.Leu206Gln (sequencing) | P | SH | chorea, dysarthria. | NA | Learning difficulties. |
| **Ferrara_2012_PIII-3** | F | 13 y | Missense, *NKX2-1*(ENST00000354822.7):c.617T>A  p.Leu206Gln (sequencing) | M | congenital-Sub H. | Chorea, dysarthria, dyspnea. | Neonatal respiratory distress. | Learning difficulties, psychosis, depression, whispering speech, self-injurious behaviors. |
| **Ferrara_2012_PIII-5** | M | 6 m | Missense, *NKX2-1*(ENST00000354822.7):c.617T>A  p.Leu206Gln (sequencing) | M | CH | NA | Neonatal respiratory distress. | NA |
| **Ferrara_2012_PIII-6** | M | 9 y | Missense, *NKX2-1*(ENST00000354822.7):c.617T>A  p.Leu206Gln (sequencing) | M | SH | Chorea, delayed motor milestones, limb weakness, dyspnea, dysarthria | NA | Learning difficulties. |
| **Ferrara_2012_PIII-7** | M | 3 y 6 m | Missense, *NKX2-1*(ENST00000354822.7):c.617T>A  p.Leu206Gln (sequencing) | M | H | Chorea, dysarthria. | Neonatal respiratory distress. | Learning difficulties. |
| **Ferrara_2012_PIII-I** | F | 4 y | Missense, *NKX2-1*(ENST00000354822.7):c.617T>A  p.Leu206Gln (sequencing) | P | SH | Chorea, limb weakness, dysarthria. | NA | Learning difficulties, epilepsy. |
| **Fons_2012_P1** | F | 6 y | Splicing, *NKX2-1*(ENST00000354822.7):c.463+1G>A/  p.? (sequencing) | D | SH | Chorea, ataxia, motor delay, gait disorder, muscular hypotonia. | pneumonia, airway infections. | NA |
| **Galambos_2010_P1** | M | 4 y 6 m | Deletion 14q13-21.3 (Karyotyping analysis) | M | Euthyroid sick syndrome, low cortisol levels. | Muscular hypotonia | Neonatal respiratory distress, chronic lung disease of prematurity/bronchopulmonary dysplasia, ductus arteriosus, foramen ovale, depressed biventricular function, | Cerebral dysgenesis, cortical dysgenesis, developmental delay. The patient passed away at 4.5 months (respiratory insufficiency and pneumonia) |
| **Gonçalves_2019_P1** | F | 6 y | Frameshift, *NKX2-1*(NM_003317.4):c.859_860insTGCC/  p.Gln287Leu*123 (sequencing) | D | CH | Chorea, muscular hypotonia, ataxia, motor delay, dysarthria, dysmetria. | NA | Chiari malformation, speech sound and coordination disorder. |
| **Gras_2012_P10_(D)** | F | 5 y | Splicing, *NKX2-1* (NM_001079668.3), c.373+1_373+4del/p.? (sequencing) | M | CpH | Chorea, muscular hypotonia | NA | Developmental delay. |
| **Gras_2012_P11_(D)** | F | 4 y | Splicing, *NKX2-1* (NM_001079668.3), c.373+1_373+4del/p.? (sequencing) | M | CpH | Chorea, muscular hypotonia | asthma | Developmental delay. |
| **Gras_2012_P13_(E)** | F | 35 y | Missense, *NKX2-1* (NM_001079668.3), c.671T>G/p.Leu224Arg (sequencing) | M | CpH | Chorea, motor tics, myoclonus | NA | Learning difficulties |
| **Gras_2012_P15_(E)** | M | 39 y | Missense, *NKX2-1* (NM_001079668.3), c.671T>G/p.Leu224Arg (sequencing) | M | CpH | Chorea, muscular hypotonia, dystonia, tremor | asthma | Learning difficulties |
| **Gras_2012_P18_(E)** | M | 8 y | Missense, *NKX2-1* (NM_001079668.3), c.671T>G/p.Leu224Arg (sequencing) | M | CpH | Chorea | asthma | Learning difficulties, ADHD. |
| **Gras_2012_P19_(E)** | F | 64 y | Missense, *NKX2-1* (NM_001079668.3), c.671T>G/p.Leu224Arg (sequencing) | NA | H | Chorea, muscular hypotonia, dystonia. | asthma | NA |
| **Gras_2012_P2_(A)** | F | 30 y | Frameshift, *NKX2-1* (NM_001079668.3), c.257dup/p. His86GlnfsTer353 (sequencing) | M | H | Chorea, muscular hypotonia, dystonia, | asthma | Learning difficulties. |
| **Gras_2012_P20_(E)** | F | 59 y | Missense, *NKX2-1* (NM_001079668.3), c.671T>G/p.Leu224Arg (sequencing) | NA | H | Chorea, muscular hypotonia, dystonia, tremor | asthma | Learning difficulties |
| **Gras_2012_P21** | F | 18 y | Frameshift, *NKX2-1* (NM_001079668.3),c.399delC/p.Met134Ter (sequencing) | NA | CpH | Chorea, muscular hypotonia | asthma | Learning difficulties |
| **Gras_2012_P22** | M | 16 y | Missense, *NKX2-1* (NM_001079668.3), c.526C>G/p.Leu176Val (sequencing) | NA | H | Chorea, muscular hypotonia, dystonia, myoclonus | asthma | Speech delay, palatal cleft, dental agenesis. |
| **Gras_2012_P23** | F | 5 y | Frameshift, *NKX2-1* (NM_001079668.3), c.786_787del/ p.Asp262GlufsTer176 (sequencing) | D | CH | Chorea, muscular hypotonia, myoclonus. | Neonatal respiratory distress | NA |
| **Gras_2012_P24** | F | 6 y | Missense, *NKX2-1* (NM_001079668.3), c.643T>G/p.Tyr215Asp(sequencing) | D | CH | Chorea | NA | NA |
| **Gras_2012_P26** | F | 16 y | Missense, *NKX2-1* (NM_001079668.3), c.719A>C/p.Gln240Pro (sequencing) | NA | CH | Chorea, muscular hypotonia | NA | NA |
| **Gras_2012_P27** | F | 20 y | Deletion 14q13.2q21.2 (6.2 Mb) (CGH array) | D | H | Chorea, muscular hypotonia | NA | Learning difficulties, ADHD. |
| **Gras_2012_P28** | F | 17 y | Deletion 14q13.3 (0.3 Mb) (CGH array) | D | CpH | Chorea, muscular hypotonia, motor and vocal tics. | NA | Learning difficulties, |
| **Gras_2012_P3_(A)** | F | 5 y | Frameshift, *NKX2-1* (NM_001079668.3), c.257dup/ p.His86GlnfsTer353 (sequencing) | NA | H | Chorea, muscular hypotonia | Lung cancer | Learning difficulties. |
| **Gras_2012_P4_(B)** | M | 12 y | Frameshift, *NKX2-1* (NM_001079668.3), c.732C>A/ p.Tyr244Ter (sequencing) | M | CpH | Chorea, muscular hypotonia | NA | Learning difficulties, ADHD |
| **Gras_2012_P7_(B)** | F | 53 y | Frameshift, *NKX2-1* (NM_001079668.3), c.732C>A/ p.Tyr244Ter (sequencing) | NA | CpH | Chorea, myoclonus | NA | NA |
| **Gu_2020_P1** | M | 2 m | Frameshift, *NKX2-1* (ENST00000354822.7):c.1124_1125insAGGTGGATAC/ p.Ser376Glyfs∗66 (sequencing) | P | CH | NA | Neonatal respiratory distress, pneumonia, dyspnea, cyanosis, interstitial lung disease, pneumonia. | Feeding difficulty, irritability, convulsions. The patient passed away at 2 months (pulmonary infections and low OxyH saturation) |
| **Hayasaka_2018_P7** | F | 11 y | Frameshift, *NKX2-1* (NM_001079668.3), c.1117C>T/p.Gln373Ter (sequencing) | NA | H | NA | pneumonia, cyanosis, ground glass opacification in lung. | NA |
| **Hayasaka_2018_P8** | M | 1 y 6 m | Frameshift, *NKX2-1* (NM_001079668.3), c.1016_1017insCCATCTCCGTGGGCAGCGG/p.Gly339fs (sequencing) | D | H | NA | interstitial lung disease, cyanosis, glass opacification in lung. | Developmental delay. |
| **Hayasaka_2018_P9** | F | 2 y 9 m | Frameshift, *NKX2-1* (NM_001079668.3), c.954_958GCAGG>  CAG/p.Gln318fs (sequencing) | D | H | NA | interstitial lung disease, pulmonary alveolar proteinosis, anomalous pulmonary venous connection, cyanosis, ground glass opacification in lung. | Developmental delay. |
| **Kharbanda_2017_P1** | F | 7 y | Deletion  14q13.2q13.3, not involving NKX2-1 (CGH array) | D | CH | Chorea, muscular hypotonia, dystonia-myoclonic movement disorder, hypermobility. | NA | Fetal finger pads, prominent forehead |
| **Kleinlein_2011_P1** | M | 10 m | Frameshift, *NKX2-1* (NM_001079668.3), c.278_308del/ p.Ala93GlyfsTer22 (sequencing) | D | Mild CH | NA | Neonatal respiratory distress, lung ground glass opacity, taquipnea, cardiac defect, vascular resistance. | The patient passed away at of 10 months (respiratory and heart failure) |
| **Koht_2016_P II:4** | F | NA | Missense, *NKX2-1* (NM_001079668.3), c.671T>G/p.Leu224Arg (NA) | M | H | Chorea, dystonia, hyperkinetic movements, delayed motor skills, | asthma | NA |
| **Koht_2016_P II:7** | F | NA | Missense, *NKX2-1* (NM_001079668.3), c.671T>G/p.Leu224Arg (NA) | M | H | Chorea, dystonia, mild ataxia, hyperkinetic movements, delayed motor skills, | asthma | NA |
| **Koht_2016_P III:6 (index)** | F | 36 y | Missense, *NKX2-1* (NM_001079668.3), c.671T>G/p.Leu224Arg (CGH array) | M | CpH | Chorea, mild ataxia, hyperkinetic movements, delayed motor skills, myoclonus. | asthma | Low verbal and memory attention |
| **Koht_2016_P III:7** | M | NA | Missense, *NKX2-1* (NM_001079668.3), c.671T>G/p.Leu224Arg (NA) | M | CpH | Hyperkinetic movements, delayed motor skills, chorea, dystonia | respiratory infections | NA |
| **Koht_2016_P IV:3** | M | NA | Missense, *NKX2-1* (NM_001079668.3), c.671T>G/p.Leu224Arg (NA) | M | CpH | Chorea, hyperkinetic movements, delayed motor skills. | asthma | Low verbal and memory attention, |
| **Krude_2002_P1** | NA | 16 y | Deletion 14q11.2q13.3 (sequencing) | NA | CH | chorea | Neonatal respiratory distress, pulmonary infections | NA |
| **Krude_2002_P2** | NA | 15 y | Missense, *NKX2-1*(NM_?) (G2626T)/p.? (sequencing) | D | CH (euthyroid hyperthyrotropinemia at 15m) | chorea | Neonatal respiratory distress, pulmonary infections | NA |
| **Krude_2002_P3** | M | 3 y | Frameshift *NKX2-1*(NM_?) (259insGG)/p.? (sequencing) | D | CH | chorea | pulmonary infections | NA |
| **Krude_2002_P4** | M | 13 y | Frameshift *NKX2-1*(NM_?), (C2519A), p.? (sequencing) | NA | CH | chorea | NA | NA |
| **Krude_2002_P5** | NA | 1 y 6 m | Frameshift *NKX2-1*(NM_?), (C1302A)/p.? (sequencing) | NA | CH | muscle hypotonia | Neonatal respiratory distress | NA |
| **Kumar_2014_P2** | M | 7 y | Missense, *NKX2-1(*ENST00000354822.7):c.701A>T/  p.Gln234Leu (sequencing) | P | H | chorea, truncal ataxia, dystonia | NA | Epilepsy, developmental delay, behavioral problems. |
| **Kumar_2014_P3** | M | 27 y | Missense, *NKX2-1(*ENST00000354822.7):c.701A>T/  p.Gln234Leu (sequencing) | NA | H | Chorea, muscular dystrophy, motor delay | pneumonia | Anxiety, learning disability, atherosclerosis. The patient passed away at the age of 27 years (cardiac arrest). |
| **Li_2023_P1** | F | 7 y | Missense, *NKX2-1* (NM_001079668.3), c.706A>G/ p.Lys236Glu (WES) | D | CH | dysarthria, ataxia, motor delay. | pneumonia | Language delay. |
| **Lynn_2020_P1** | M | 7 m | Deletion 14q13.1-14q21.1 (CGH array) | NA | CH | Muscular hypotonia | Neonatal respiratory distress, pulmonary infections, pulmonary hypertension, tachypnea, hypoxemia, chronic respiratory failure | Neurodevelopmental delay, feeding dysfunction. |
| **Makretskaya_2018_P74** | NA | NA | Deletion, NKX2-1 (NM_001079668.3):chr14:36.986.917-36.987.061 or c.628_772del (NGS) | NA | CH | chorea | NA | NA |
| **Makretskaya_2018_P75** | NA | NA | Missense, *NKX2-1* (NM_001079668.3), c.1180A>G/ p.Thr394Ala (NGS) | NA | CH | NA | NA | NA |
| **Maric_2020_P1** | F | 5 y | Frameshift*, NKX2-1*(NM_003317.4):c.254dupG/  p.Tyr86Leu*323 (sequencing) | D | SH | Chorea, dystonia, muscular hypotonia, psychomotor delay. | Neonatal respiratory distress, pulmonary hemorrhage, pulmonary hypertension, respiratory infections, pneumonia. | NA |
| **Moya _2018_P1** | M | NA | Frameshift, *NKX2-1*(NM_001079668.3), c.223dupG/p.Val75Glyfs*334  (sequencing) | D | CH | chorea, muscular hypotonia, psychomotor delay | Tachypnoea, intercostal respiratory retractions, poor peripheral perfusion, cyanosis, lung diffuse ground glass opacity, hyaline membrane disease | Flaring nostrils, gastroesophageal reflux. |
| **Nagasaki_2008_P1** | M | NA | Frameshift, NKX2-1(NM_003317.4): c.470_479delinsGCG/p. P157fsX196 (sequencing) | D | CH (resistance to TSH) | chorea, muscular hypotonia, | respiratory infections | Developmental delay. |
| **Nattes_2017_P1** | M | 5 y | Frameshift, *NKX2-1*(NM_001079668.3), c.714G>A/ p.Trp238Ter (sequencing) | NA | H | Chorea, muscular hypotonia | Neonatal respiratory distress | NA |
| **Nattes_2017_P10** | F | 42 y | Frameshift, *NKX2-1*(NM_001079668.3), c.267dup/ p.His90AlafsTer349  (sequencing) | NA | Gestational H | chorea | infiltrative lung disease, lung fibrosis | NA |
| **Nattes_2017_P11** | F | 28 y | Splicing, *NKX2-1*(NM_001079668.3), c.463+2T>C/p.? (sequencing) | NA | H | chorea | infiltrative lung disease, respiratory insufficiency | NA |
| **Nattes_2017_P14** | M | 19 y | Deletion 14q13q13 (FISH) | NA | H | Chorea, Muscular hypotonia, psychomotor delay | Neonatal respiratory distress | NA |
| **Nattes_2017_P2** | M | 9 m | Frameshift, *NKX2-1*(NM_001079668.3), c.714G>A/p. Trp238Ter (sequencing) | NA | H | Muscular Hypotonia, psychomotor delay | Neonatal respiratory distress | NA |
| **Nattes_2017_P3** | M | 3 y | Frameshift, *NKX2-1*(NM_001079668.3), c.344dup/ p.Tyr116LeufsTer323 (sequencing) | NA | H | Chorea, Muscular hypotonia, psychomotor delay | Neonatal respiratory distress | NA |
| **Nattes_2017_P4** | F | 1 y 6 m | Missense, NKX2-1 (NM_001079668.3), c.583C>T/p. Arg195Trp (sequencing) | NA | CpH | Muscular hypotonia | Neonatal respiratory distress | The patient passed away at 18 months (respiratory failure). |
| **Nattes_2017_P5** | F | 10 y | Frameshift, *NKX2-1*(NM_001079668.3), c.876_877del/ p.Leu293GlyfsTer145 (sequencing) | NA | H | Chorea, Muscular hypotonia, psychomotor delay | Neonatal respiratory distress | NA |
| **Nattes_2017_P6** | M | 2 y | Splicing, *NKX2-1*(NM_001079668.3), c.463+2T>C/p.? (sequencing) | NA | H | Chorea, Muscular hypotonia, psychomotor delay | Neonatal respiratory distress | NA |
| **Parnes_2019_P1** | M | 8 y | Frameshift, *NKX2-1*(NM_001079668.3),c.754_755insT /p.Asp252ValfsTer187 (WES) | D | H | Chorea, Muscular Hypotonia | Neonatal respiratory distress, respiratory infections | OCD, ADHD, developmental delay, stuttering |
| **Parnes_2019_P2** | F | 2 y | Frameshift, *NKX2-1*(NM_001079668.3),c.390C>G / Tyr130Ter (WES) | M | H | Chorea, Muscular Hypotonia | Neonatal respiratory distress, pulmonary hypertension | Developmental delay. |
| **Parnes_2019_P3** | F | 31 y | Frameshift, *NKX2-1*(NM_001079668.3),c.390C>G/ Tyr130Ter (WES) | D | H | Chorea, Muscular hypotonia, dystonia, myoclonus | Neonatal respiratory distress | Developmental delay. |
| **Parnes_2019_P5** | M | 7 y | Frameshift, *NKX2-1*(NM_001079668.3),c.397dup/ p.Thr133AsnfsTer306 (sequencing) | NA | H | Muscular Hypotonia, chorea | NA | Epilepsy, developmental delay. |
| **Prasad_2019_P1** | F | 4 y | Deletion 14q13.2-q21.1 (35,975, 495–40,890, 854) (4,9Mb) Deletion 3p12.3-p13 73, (893, 402–74, 297, 269) (CGH array) | NA | CH, hyperinsulinism, growth hormone deficiency, cortisol deficiency, undetectable gonadotrophins. | Chorea | Neonatal respiratory distress, pulmonary hypertension, ductus arteriosus, pulmonary infection | NA |
| **Provenzano_2016_P1** | F | 32 y | Missense, *NKX2-1*(NM_003317.4): c.516G>T/  p.Gln172His (sequencing) | NA | H | chorea, ataxia, hyperkinetic movements, dysdiadochokinesia, dysmetria, myoclonic jerks, myoclonus. | NA | Mild cerebellar atrophy, glioblastoma. |
| **Provenzano_2016_P2** | M | 12 y | Missense, *NKX2-1*(NM_003317.4): c.623G>C/  p.Trp208Ser (sequencing) | NA | H | chorea, hyperkinetic movements. | NA | NA |
| **Safi_2017_PII-2** | F | NA | Splicing, *NKX2-1(*ENST00000354822.7):c.464-9C>A/  p.? (NA) | D | CH | Gait ataxia, tremor | Neonatal respiratory distress | NA |
| **Safi_2017_PII-3** | F | NA | Splicing, *NKX2-1(*ENST00000354822.7):c.464-9C>A/  p.? (NA) | D | H | NA | NA | Bipolar disorder, cognitive impairment. |
| **Safi_2017_PIII-1** | F | NA | Splicing, *NKX2-1(*ENST00000354822.7):c.464-9C>A/  p.? (NA) | M | CH | NA | Neonatal respiratory distress, respiratory infections, interstitial lung disease | Developmental delay. |
| **Safi_2017_PIII-2** | F | NA | Splicing, NKX2-1(ENST00000354822.7):c.464-9C>A/ | M | CH | NA | bronchiolitis, pneumonia, interstitial lung disease | Developmental delay. |
| **Salerno_2014_P1** | M | 28 d | Frameshift, *NKX2-1*(NM_001079668.3), c.424G>T/ p.Gly142Ter (sequencing) | P | CH | Muscular hypotonia | Neonatal respiratory distress, tachypnoea, ground glass areas in lungs. | NA |
| **Salvado_2013_P1** | F | 19 y | Frameshift, *NKX2-1*(NM_001079668.3), c.915del/ p.Ala306ArgfsTer75 (NA) | M | CH | chorea, motor impairment, dyskinesia. | Neonatal respiratory distress. | Intellectual impairment. |
| **Salvado_2013_P2** | F | 13 y | Frameshift, *NKX2-1*(NM_001079668.3), c.915del/ p.Ala306ArgfsTer75 (NA) | M | CH | chorea, ataxia, dyskinesia, motor impairment. | Neonatal respiratory distress. | Intellectual impairment. |
| **Salvado_2013_P3** | F | 43 y | Frameshift, *NKX2-1*(NM_001079668.3), c.915del/ p.Ala306ArgfsTer75 (NA) | M | Gestational H | motor impairment | NA | Poliomyelitis |
| **Salvatore_2010_P1** | F | 26 y | Frameshift, *NKX2-1*(NM_001079668.3), c.524C>A/ p.Ser175Ter (sequencing) | P | H | Chorea. | ventricular dilatation | Psychosis, memory deficit, brain hypometabolism. |
| **Salvatore_2010_P2** | M | 56 y | Frameshift, *NKX2-1*(NM_001079668.3), c.524C>A/ p.Ser175Ter (sequencing) | NA | H | Chorea, hyperkinesia, apraxia, | ventricular dilatation | Development delay, memory deficit, brain hypometabolism. |
| **Salvatore_2010_P3** | M | 5 y | Frameshift, *NKX2-1*(NM_001079668.3), c.524C>A/ p.Ser175Ter (sequencing) | M | CH | chorea. | Neonatal respiratory distress | Developmental delay congenital anomalies (reflux with pyelectasis and megabladder, patent foramen ovale). |
| **Santos-Silva_P10** | F | 7 y | Frameshift, *NKX2-1*(NM_001079668.3),c.859_860insTGCC/  p.Arg287Leufs*  (sequencing) | D | CH | Chorea, hypotonia, ataxia, dysmetria. | NA | Developmental delay. |
| **Shiohama_2018_P1** | M | 12 y 6 m | Deletion 14q13.2q13.34 (35,352,962–36,903,832)x1 dn (CGH array) | NA | Asymptomatic H | chorea, muscular hypotonia, drop attacks. | NA | Developmental delay. |
| **Tanaka_2020_P26** | M | 11 y | Frameshift, *NKX2-1*(NM_001079668.3), c.596C>A/ p.Ser199Ter (sequencing) | NA | CH | NA | NA | NA |
| **Thust_2022_F1_P1** | NA | 49 y | Missense, (NM_003317.4): c.631A>T/  p.(Asn211Tyr) (sequencing) | D | H | chorea, ataxia, dysdiadochokinesia. | NA | Development delay, CSF-filled sella turcica in pituitary gland. |
| **Trevisani_2022_P1** | M | 1y 6 m | Frameshift, *NKX2-1*(NM_001079668.2) c.872C>G/Prol219ARg (sequencing) | D | SH, growth hormone and serum insulin-like growth factor-I deficiency. | Hypotonia, dystonia, ligamentous hyperlaxity, developmental delay. | Asthma, tracheal-laryngomalacia, respiratory infections. | Otitis media, hippocampus dysmorphism. |
| **Uematsu_2012_P1** | F | 7 y | Deletion 14q12-13 (2.6 Mb) (CGH array) | D | CH | Chorea, gait disturbances, muscular hypotony. | NA | Development delay, reduced blood flow caudate nuclei. |
| **Uematsu_2012_P2** | F | 5 y | Missense, *NKX2-1*(NM_?), c.613G>T/  p.V205P (sequencing) | M | CH | Chorea, gait disturbances, muscular hypotony. | NA | Development delay, reduced blood flow caudate nuclei. |
| **Uematsu_2012_P3** | M | 6 y | Missense, *NKX2-1*(NM_?), c.613G>T/  p.V205P (sequencing) | M | H | Chorea, gait disturbances, muscular hypotony. | NA | Development delay, reduced blood flow caudate nuclei. |
| **Veneziano_2014_P II05 (proband)** | F | 49 y | Frameshift, *NKX2-1*(ENST00000354822.7):c.631A>T/  p.Lys211Ter (sequencing) | D | H, macroadenoma, cystic pituitary mass. | Chorea, ataxia, dysarthria, dysdiadochokinesia, | NA | NA |
| **Veneziano_2014_P III01** | F | 26 y | Frameshift, *NKX2-1*(ENST00000354822.7):c.631A>T/  p.Lys211Ter (sequencing) | M | H, low prolactin level, cystic pituitary mass. | Chorea, ataxia. | chest infections | NA |
| **Villafuerte_2018_P1** | F | 10 y | Deletion14q13.2-q21.1 (3.32 Mb) (CGH array) | D | CH | Chorea, muscular Hypotonia, dysplasia, joint hyperlaxity. | Neonatal respiratory distress, respiratory infections | Learning delay. |
| **Villamil-Osorio_2021_P1** | M | NA | Deletion 14q12-q21.1 (14.7 Mb) (CGH array) | NA | H | NA | lung sepsis, interstitial lung disease and hypertension | Epilepsy, microcephaly, corpus callosum dysgenesis, supratentorial leukoencephalopathy. |

**Abbreviations**

**Sex^a^.**

M=male

F=female

NA=not available

**Age^b^.**

d=days

m=months

y=years

NA=not available

**gene test^c^.**

NKX2-1 gene sequencing

CGH= Comparative Genome Hybridization

FISH= fluorescence in situ hybridization

NGS= Next-Generation DNA Sequencing

WES= Whole Exome DNA Sequencing

Karyotyping analysis

NA= not available

**Inherited^d^.**

D=de novo

M=maternal

P=paternal

NA= not available

**Endocrine alterations^e^**

H=hypothyroidism

CH=congenital hypothyroidism

SH=subclinical hypothyroidism

CpH=compensated hypothyroidism

**Note:** Data extraction was conducted by BCH and JDOE on January 15, 2024. The studies listed in this table were confirmed to be eligible for inclusion in the review. All necessary data were extracted from each study included in the systematic review and/or meta-analysis to ensure that the analyses can be replicated.
