# Supplementary material for: Systematic review of thyroid function in NKX2-1-related disorders: Treatment and follow-up
Source: PLoS One. 2024 Oct 28;19(10):e0309064. doi: 10.1371/journal.pone.0309064 (PMC11515955; doi:10.1371/journal.pone.0309064)
Supplement: S5 File — Levothyroxine (LT4) treatment strategy for hypothyroidism at the patient level. This table exclusively presents data for patients who underwent LT4 treatment. (DOCX) [file pone.0309064.s006.docx]

**S5. LT4 treatment strategy of patients with *NKX2-1*-RD.** Levothyroxine (LT4) treatment strategy for hypothyroidism at the patient level. This table exclusively presents data for patients who underwent LT4 treatment.

| **Patient and reference** | **Type of hypothyroidism^a^** | **Confirmation of hypothyroidism** | **Onset age of LT4 initiation and dosage** | **Follow-up** |
| --- | --- | --- | --- | --- |
| **Barreiro_2011_P1** | CH | Altered neonatal screening and corroborated by later diagnostic tests. TSH concentration of 8.3 mU/L (TSH threshold 8.2 mlU/L). | 8^th^ day of life, 14 μg/kg/day | The dose was successively adjusted to progressive normalization of TSH levels. |
| **Doyle_2004_P III2** | CH | Altered neonatal screening and corroborated by later diagnostic tests.  Screening: TSH 49 µU/ml, T4 normal.  Confirmatory: TSH 17 µU/ml, T4 8.4 µg/dl. | 35^th^ day of life,  37.5 µg/day | The dose was successively increased to 62.5 µg/day (at 10 years) |
| **Doyle_2004_P III3** | CH | Altered neonatal screening and corroborated by later diagnostic tests.  Screening TSH 39 µU/ml. T4 normal.  Confirmatory: TSH 24.5 µU/ml, T4 6.3 µg/dl. | 19^th^ day of life,  37.5 µg/day | The dose was successively increased to 62.5 µg/day (at 4 years) |
| **Fons_2012_P1** | SH | Normal neonatal screening and altered later diagnostic tests.  At 2 years of age, she was diagnosed with subclinical hypothyroidism. TSH: 16.36 mU/L (normal range: 0.3–4.5); FT4: 15.3 pmol/L (normal range: 9.1–25), with normal thyroid peroxidase antibody and thyroid ultrasound exam. | 2 years, 50 μg/day | The dose was gradually diminished due to progressive normalization of TSH levels |
| **Gonçalves_2019_P1** | CH | Altered neonatal screening and later diagnostic tests.  TSH level of 110.9 mU/L | 8^th^ day of life, 25 μg/d | Follow-up at 24 months. NA about LT4 dosage. |
| **Kharbanda_2017_P1** | CH | Altered neonatal screening and corroborated by later diagnostic tests. Mild capillary TSH elevation was detected on newborn screening (14 and 10.5 mU/l on initial and repeat testing). Venous TSH was elevated at 25 mU/l (reference range 0.72-13.1), FT4 was normal at 15.5 pmol/l (normal range 9-26). | Age at onset of LT4 treatment not available, she received 25 μg/day at onset. | The dose was stopped because of thyroid function normalization (at 4 years) |
| **Moya _2018_P1** | CH | Altered neonatal screening and corroborated by later diagnostic tests.  Filter paper TSH values of 70 mU/L (normal, 10 mlU/L), confirmed in serum samples on the 10^th^ day of life (TSH: 224 mU/L; and FT4: 0.6 ng/dL). | 10^th^ day of life, 37.5 μg/d | Thyroid function normalization at day 30. The dose was gradually increased to 50 µg/day at 3 months and 100 µg/day at 14 years. |
| **Nagasaki_2008_P1** | CH (resistance to TSH) | Altered neonatal screening and corroborated by later diagnostic tests  Neonatal screening: borderline TSH level 9.1 mU/L (upper limit of normal for age 8 mU/L.  1 month of age: normal TSH 1.2 mU/L, normal T4 10.9 μg/dl  4 months of age: normal TSH 41.8 mU/L, elevated FT4 1.7 ng/dl). | Onset 4 months, dosage not available. At 14 years of age, 100 μg/day | At 14 years, TSH levels increased due to LT4 discontinuance for 4 weeks. Exaggerated TSH response. |
| **Provenzano_2016_P1** | H | No neonatal screening. Later confirmatory diagnostic tests. | 18 months, 125 μg/day | Highly increased levels of TSH. |
| **Shiohama_2018_P1** | Asymptomatic H | No neonatal screening. Later confirmatory diagnostic tests.  At the age of 3 years and 6 months, he was diagnosed with asymptomatic hypothyroidism (FT4 1.10 ng/dl (normal range 0.7–1.48 ng/dl), TSH 10.96 μU/ml (normal range 0.35–4.94 μU/ml) | 4 years , 2 μg/kg/d | At 11 years and 8 months, LT4 discontinuation due to euthyroidism, but frequent drop attacks were present. After LT4 restoration (1 µg/kg/day), the drop attacks were resolved. Euthyroidism was maintained even when treatment was suspended. |
| **Tanaka_2020_P26** | CH | Altered neonatal screening and corroborated by later diagnostic tests. (Initial evaluation TSH: 29.6 μU/ml, FT4: 0.7 ng/dl). | 21 days of life – dosage available at 3 years of age 30 μg/day = 1 μg/kg/day | 1µg/kg/day at discontinuation. |
| **Trevisani_2022_P1** | SH | No neonatal screening. Later confirmatory diagnostic tests.  18 months of age: Slightly higher TSH levels (5.89 μU/mL; normal range 0.50–4.50 μU/mL) associated with normal levels FT4 (11.1 pg/mL; normal range 8–18 pg/mL) (suggesting subclinical hypothyroidism state). | 21 months of age, 0.88 μg/kg/day | The dose was successively increased to 1.06 µg/kg/day (at 18 months). Normalization of TSH levels. |

**Abbreviations**

**LT4:** levothyroxine

**TSH:** thyroid stimulating hormone.

**T4:** thyroxine

**FT4:** free thyroxine

**Type of hypothyroidism^a^**

**H**=hypothyroidism

**CH**=congenital hypothyroidism

**SH**=subclinical hypothyroidism

**Note:** Data extraction was conducted by BCH and JDOE on January 15, 2024. The studies listed in this table were confirmed to be eligible for inclusion in the review. All necessary data were extracted from each study included in the systematic review and/or meta-analysis to ensure that the analyses can be replicated.
