# Supplementary material for: Systematic review of thyroid function in NKX2-1-related disorders: Treatment and follow-up
Source: PLoS One. 2024 Oct 28;19(10):e0309064. doi: 10.1371/journal.pone.0309064 (PMC11515955; doi:10.1371/journal.pone.0309064)
Supplement: S6 File — Presentation of the quality assessment of included references for the treatment and follow-up of endocrine diseases in patients with NKX2-1-RD. Table A. Quality assessment of the case reports and case series included in this study. Table B. Quality assessment of the cohort study included in this study. (DOCX) [file pone.0309064.s007.docx]

**S6. Quality assessment of included references.** Presentation of the quality assessment of included references for the treatment and follow-up of endocrine diseases in patients with *NKX2-1*-RD.

**Table A.** Quality assessment of the case reports and case series included in this study.

|  |  | **Case series/** | | **Case reports** | | | |  |  |  |
| --- | --- | --- | --- | --- | --- | --- | --- | --- | --- | --- |
| **Domain** | **Selection** | **Ascertainment** | | **Causality** | | | | **Reporting** | **Final score** | **Quality** |
|  | **1** | **2** | **3** | **4** | **5** | **6** | **7** | **8** |  |  |
| **Asmus_2005** | 0 | 0 | 1 | 0 | 0 | 0 | 1 | 0 | 2 | **Poor** |
| **Balicza_2018** | 0 | 0 | 1 | 0 | 0 | 0 | 1 | 0 | 2 | **Poor** |
| **Barnett_2012** | 0 | 0 | 1 | 0 | 0 | 0 | 1 | 0 | 2 | **Poor** |
| **Barreiro_2011** | 0 | 1 | 1 | 0 | 0 | 1 | 1 | 0 | 4 | **Medium** |
| **Carré_2009** | 0 | 0 | 1 | 0 | 0 | 0 | 1 | 0 | 2 | **Poor** |
| **Delestrain_2023** | 0 | 0 | 1 | 0 | 0 | 0 | 1 | 0 | 2 | **Poor** |
| **Doyle_2004** | 0 | 0 | 1 | 0 | 0 | 0 | 1 | 0 | 2 | **Poor** |
| **Ferrara_2012** | 0 | 0 | 1 | 0 | 0 | 0 | 1 | 0 | 2 | **Poor** |
| **Fons_2012** | 0 | 1 | 1 | 0 | 0 | 0 | 1 | 0 | 3 | **Medium** |
| **Galambos_2010** | 0 | 0 | 1 | 0 | 0 | 0 | 1 | 0 | 2 | **Poor** |
| **Gonçalves_2019** | 1 | 1 | 1 | 0 | 0 | 0 | 1 | 0 | 4 | **Medium** |
| **Gras_2012** | 0 | 0 | 1 | 0 | 0 | 0 | 1 | 0 | 2 | **Poor** |
| **Gu_2020** | 0 | 0 | 1 | 0 | 0 | 0 | 0 | 0 | 1 | **Poor** |
| **Hayasaka_2018** | 1 | 0 | 1 | 0 | 0 | 0 | 0 | 0 | 2 | **Poor** |
| **Kharbanda_2017** | 0 | 1 | 1 | 0 | 0 | 1 | 1 | 0 | 4 | **Medium** |
| **Kleinlein_2010** | 0 | 0 | 1 | 0 | 0 | 0 | 1 | 0 | 2 | **Poor** |
| **Koht_2016** | 0 | 0 | 1 | 0 | 0 | 0 | 1 | 0 | 2 | **Poor** |
| **Krude_2022** | 0 | 0 | 1 | 0 | 0 | 0 | 1 | 0 | 2 | **Poor** |
| **Kumar_2014** | 0 | 0 | 1 | 0 | 0 | 0 | 1 | 0 | 2 | **Poor** |
| **Li_2023** | 0 | 0 | 1 | 0 | 0 | 0 | 1 | 0 | 2 | **Poor** |
| **Lynn_2020** | 0 | 0 | 1 | 0 | 0 | 0 | 1 | 0 | 2 | **Poor** |
| **Maric_2020** | 0 | 0 | 1 | 0 | 0 | 0 | 1 | 0 | 2 | **Poor** |
| **Moya _2018** | 0 | 1 | 1 | 0 | 0 | 1 | 1 | 1 | 5 | **Good** |
| **Nagasaki_2008** | 0 | 1 | 1 | 0 | 0 | 1 | 1 | 0 | 4 | **Medium** |
| **Nattes_2017** | 1 | 0 | 1 | 0 | 0 | 0 | 1 | 0 | 3 | **Medium** |
| **Parnes_2019** | 1 | 0 | 1 | 0 | 0 | 0 | 0 | 0 | 2 | **Poor** |
| **Prasad_2019** | 0 | 0 | 1 | 0 | 0 | 0 | 1 | 0 | 2 | **Poor** |
| **Provenzano_2016** | 0 | 0 | 1 | 0 | 0 | 0 | 1 | 0 | 2 | **Poor** |
| **Safi_2017** | 0 | 0 | 1 | 0 | 0 | 0 | 1 | 0 | 2 | **Poor** |
| **Salerno_2014** | 0 | 0 | 1 | 0 | 0 | 0 | 0 | 0 | 1 | **Poor** |
| **Salvado_2013** | 0 | 0 | 1 | 0 | 0 | 0 | 1 | 0 | 2 | **Poor** |
| **Salvatore_2010** | 0 | 0 | 1 | 0 | 0 | 0 | 1 | 0 | 2 | **Poor** |
| **Santos-Silva_2019** | 1 | 0 | 1 | 0 | 0 | 0 | 1 | 0 | 3 | **Medium** |
| **Shiohama_2018** | 0 | 1 | 1 | 0 | 1 | 1 | 1 | 1 | 6 | **Good** |
| **Tanaka_2020** | 1 | 1 | 1 | 0 | 0 | 1 | 1 | 0 | 5 | **Good** |
| **Thust_2022** | 0 | 0 | 1 | 0 | 0 | 0 | 0 | 0 | 1 | **Poor** |
| **Trevisani_2022** | 0 | 1 | 1 | 0 | 0 | 1 | 1 | 0 | 4 | **Medium** |
| **Uematsu_2012** | 0 | 0 | 1 | 0 | 0 | 0 | 1 | 0 | 2 | **Poor** |
| **Veneziano_2014** | 0 | 0 | 1 | 0 | 0 | 0 | 1 | 0 | 2 | **Poor** |
| **Villafuerte_2018** | 0 | 0 | 1 | 0 | 0 | 0 | 1 | 0 | 2 | **Poor** |
| **Villamil-Osorio_2021** | 0 | 0 | 1 | 0 | 0 | 0 | 1 | 0 | 2 | **Poor** |

**Table B.** Quality assessment of the cohort study included in this study.

|  | **Cohort studies** | | | |  |  | | |
| --- | --- | --- | --- | --- | --- | --- | --- | --- |
|  | **Selection** | | | | **Comparability** | **Outcome** | | |
| **Category** | **Representativeness of the exposed cohort** | **Selection of the non-exposed cohort** | **Ascertainment of exposure** | **Demonstration that outcome of interest was not present at start of study** | **Comparability of cohorts on the basis of the design or analysis** | **Assessment of outcome** | **Was follow-up long enough for outcomes to occur** | **Adequacy of follow up of cohorts** |
| **Makretskaya_2018** |  |  |  |  |  |  |  |  |

**Note:** Data extraction was conducted by BCH and JDOE on January 15, 2024. The studies listed in this table were confirmed to be eligible for inclusion in the review. All necessary data were extracted from each study included in the systematic review and/or meta-analysis to ensure that the analyses can be replicated.
